# Supplementary material for: Vortex phase matching as a strategy for schooling in robots and in fish
Source: Nat Commun. 2020 Oct 26;11:5408. doi: 10.1038/s41467-020-19086-0 (PMC7588453; doi:10.1038/s41467-020-19086-0)
Supplement: Supplementary file 1 — Supplementary Information [file 41467_2020_19086_MOESM1_ESM.pdf]

Supplementary Information

Vortex phase matching as a strategy for schooling in robots and in fish

Li *et.al*

Supplementary Figures

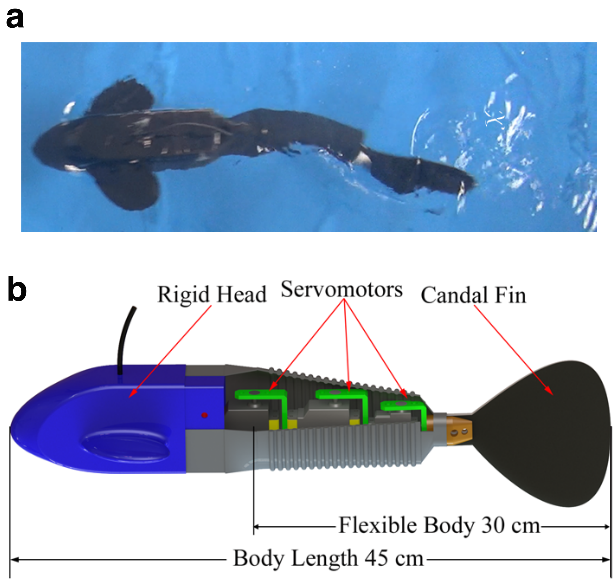

Supplementary Fig. 1: **The bio-mimetic robotic fish model.** (**a**) A snapshot of the robotic fish swimming freely. (**b**) Illustration of the inner structure of the robotic fish.

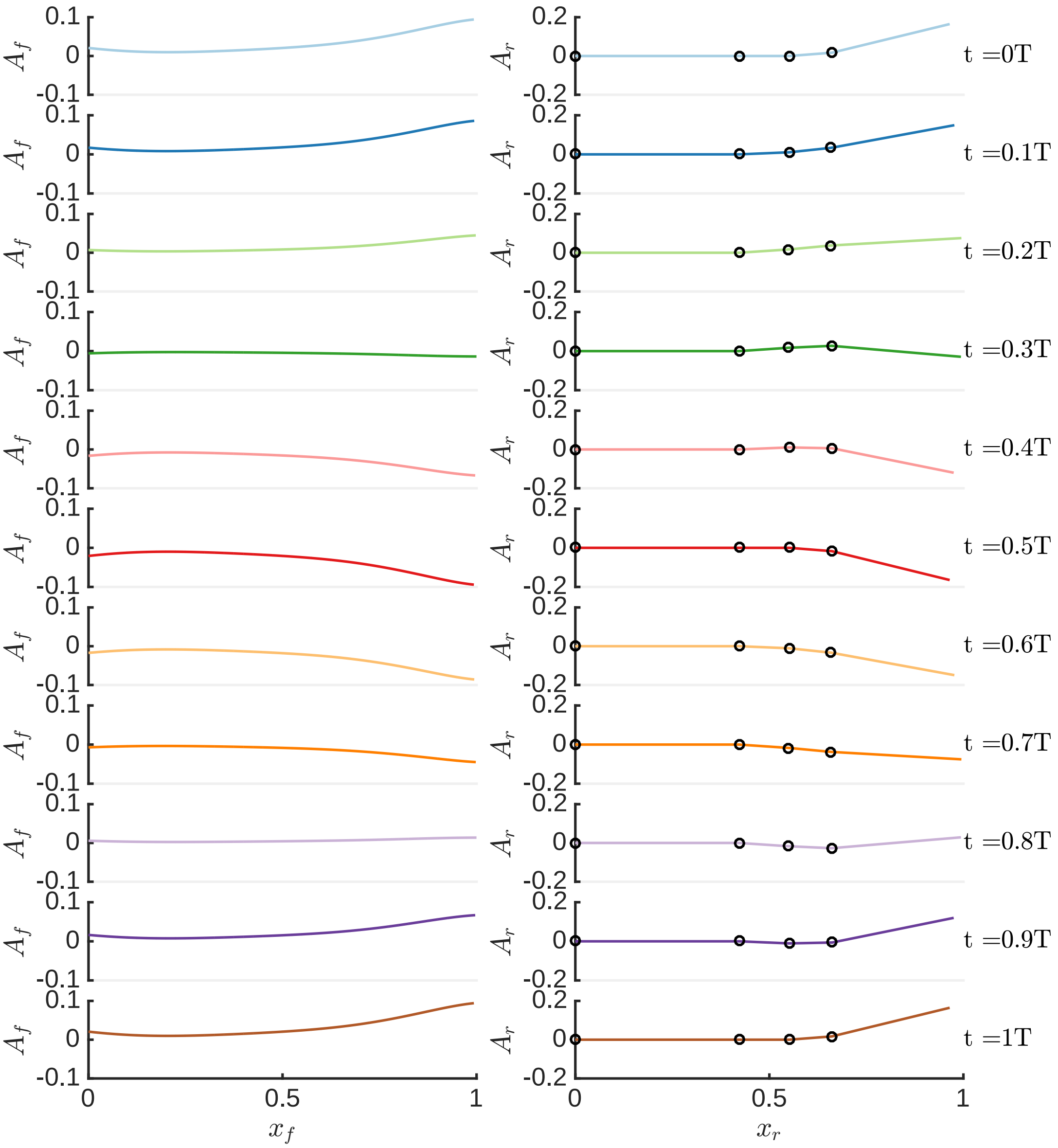

Supplementary Fig. 2: **The kinematic setting for the robotic fish.** The kinematics of the robotic fish body is determined according to Fig. 4 in Reference [1]. Left column shows the kinematics plotted according to the values in the reference. Right column shows the kinematics of the robot with three joints represented with open circles. Kinematic parameters are shown in the Supplementary Note 1.

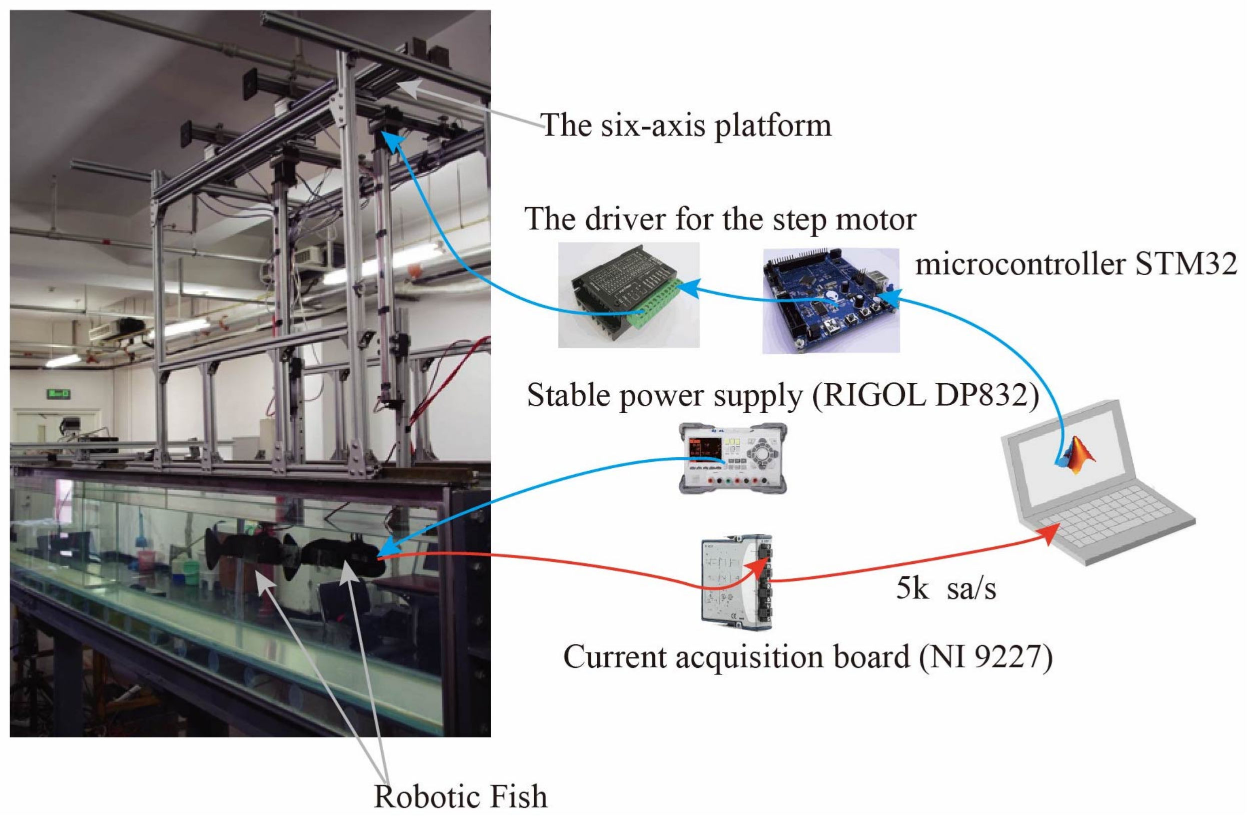

Supplementary Fig. 3: **A snapshot and the schematic of the test platform.** To control the spatial position of the two robots, a computer first sends the commands to the microcontroller STM32. And then the microcontroller sends signals to the driver to control the step motors, thus the position of the robot. The electrical current values are acquired by a National Instruments (NI 9227) at 5000 samples per second.

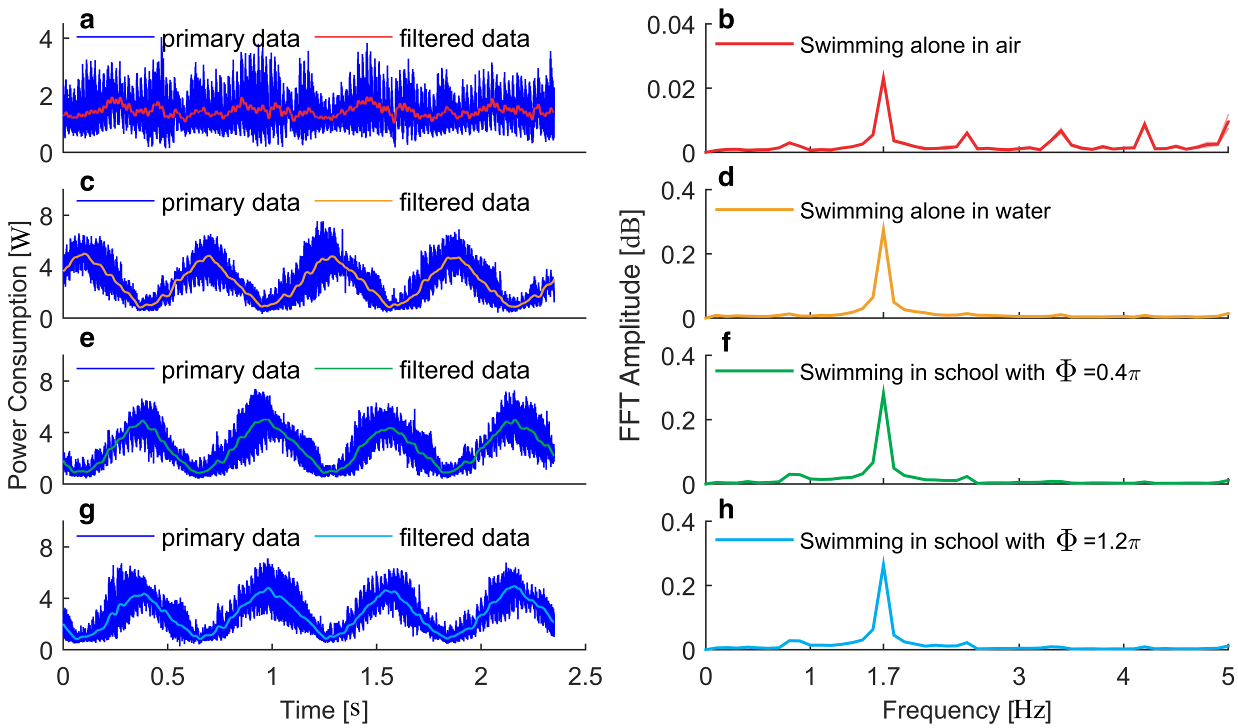

Supplementary Fig. 4: **Measurement and verification of the power consumption of the robot.** Power values of fish swimming alone in the air (**a, b**), alone in the water (**c, d**), in a school with destructive vortex phase interaction (**e, f**) and in a school with beneficial vortex phase interaction (**g, h**). Time domain plots of the power values from our power detection system are shown in (**a, c, e, g**). The power values are filtered by a Kalman filter. Frequency domain plots of the power values are shown in (**b, d, f, h**). The frequency corresponds to the double value of the undulating frequency. The robot is controlled by a CPG controller. The tail moves as a sinusoidal function, therefore, the moving speed of the tail tip will also be a sinusoidal function. This will also lead the power cost changing periodically. The main frequency of the power (1.7 Hz) is double of that of the fish swimming (0.85 Hz). This is due to the power cost of robotic fish being symmetrically between moving from leftmost to rightmost and from rightmost to leftmost.

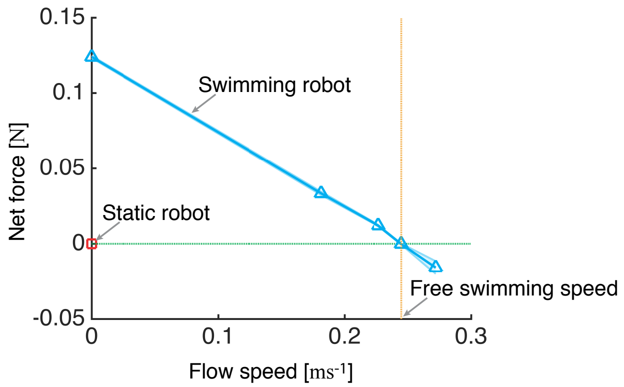

Supplementary Fig. 5: **Net force exerted on the robotic fish in the flow tank.** The net force — measured for the flow speed that was set by the speed of the free swimming robot, and which was used in the power consumption study — equals 0. Error bars are standard error of the mean and some are smaller than the symbol size.

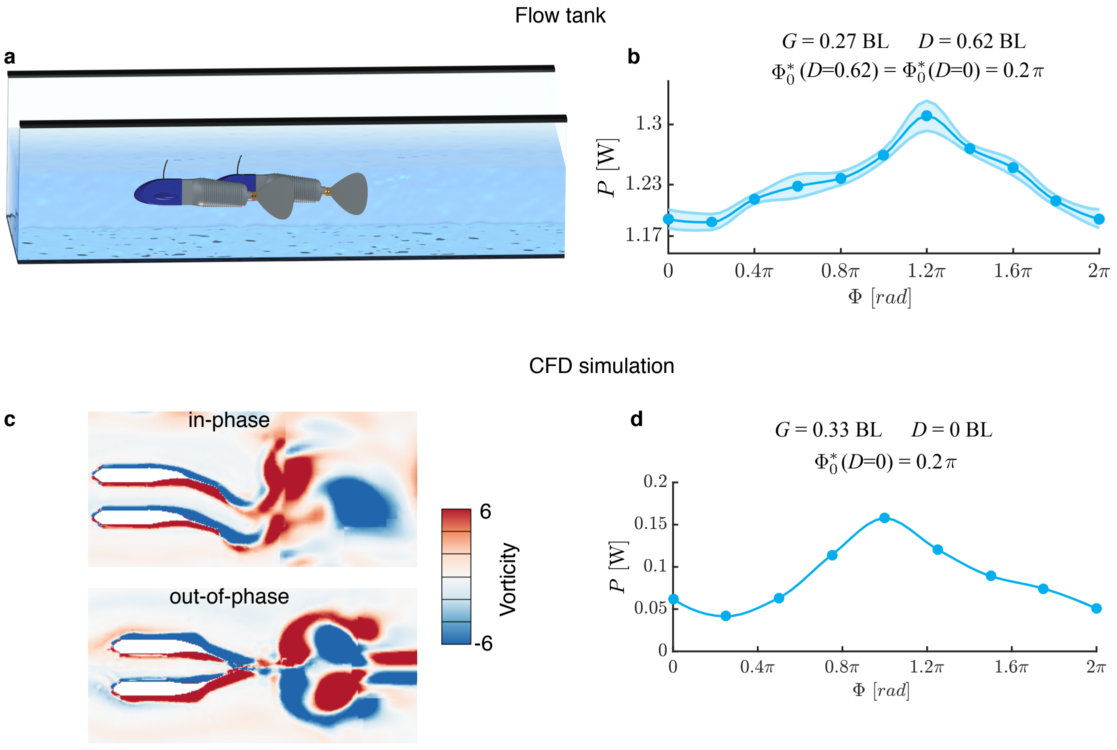

Supplementary Fig. 6: **Optimal phase differences for the follower in flow tank experiment (a) and two dimensional (2D) computational fluid dynamics (CFD) simulation (c)** (see Supplementary Note 2). (**b,d**) In both cases the power costs have an approximately sinusoidal relationship with phase difference Φ. For the applied left-right distances G, the optimal initial phase differences, Φ_0^*, that minimise the follower's power cost are both around 0.2π. Error bars are standard error of the mean. (**b**) Experiments are conducted with front-back distance D at 0.62 BL. According to our VPM rule, this gives similar hydrodynamic interactions as a function of phase difference as when D equals 0. (**d**) Simulations are conducted with front-back distance D at 0 BL, left-right distance G was set slightly larger as compare to that in the experiments to avoid grid/mesh collisions around the tailtip.

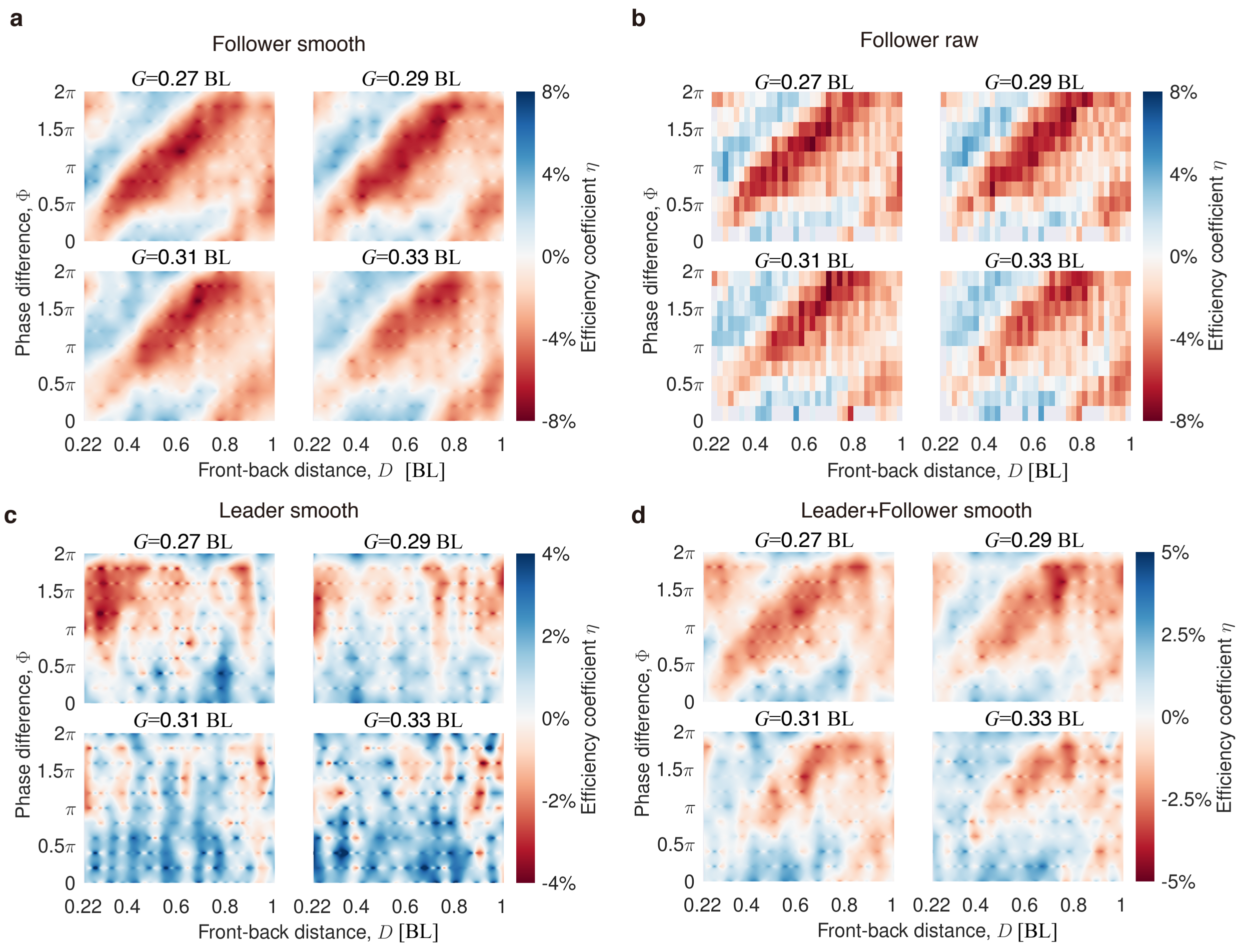

Supplementary Fig. 7: **Efficiency coefficient η of the follower, leader, and follower and leader (follower+leader).** (**a**) Linear correlations between Φ and D are clear for the follower regardless of G. (**b**) Corresponding raw data. This linear correlation disappears for the leader (**c**) which indicates that energy saving for the leader is possible, but complex. (**d**) Considering the leader and follower as a whole system, there is a weaker linear correlation indicating that the follower saves more energy than does the leader.

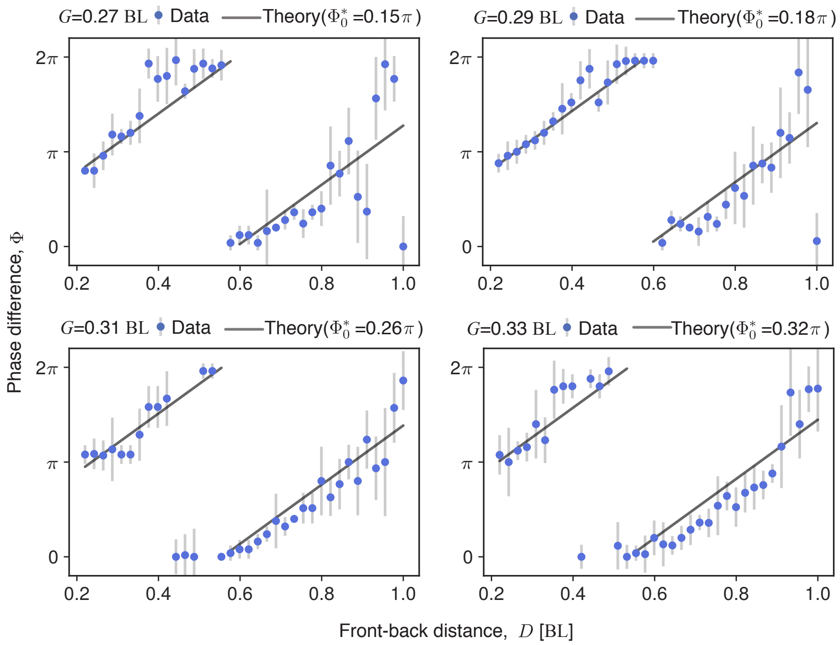

Supplementary Fig. 8: **Optimal initial phase difference Φ_0^* values for different left-right distances for the robotic fish experiments.** The point of maximal energy saving for a given front-back distance was determined for all 4 left-right distances (G=0.27, 0.29, 0.31 and 0.33 BL). The grey lines depict the theoretical predictions, which are calculated based on the vortex phase matching equation (Equation 2) with Φ_0^* determined by periodic least square fitting. Error bars are standard error of the mean.

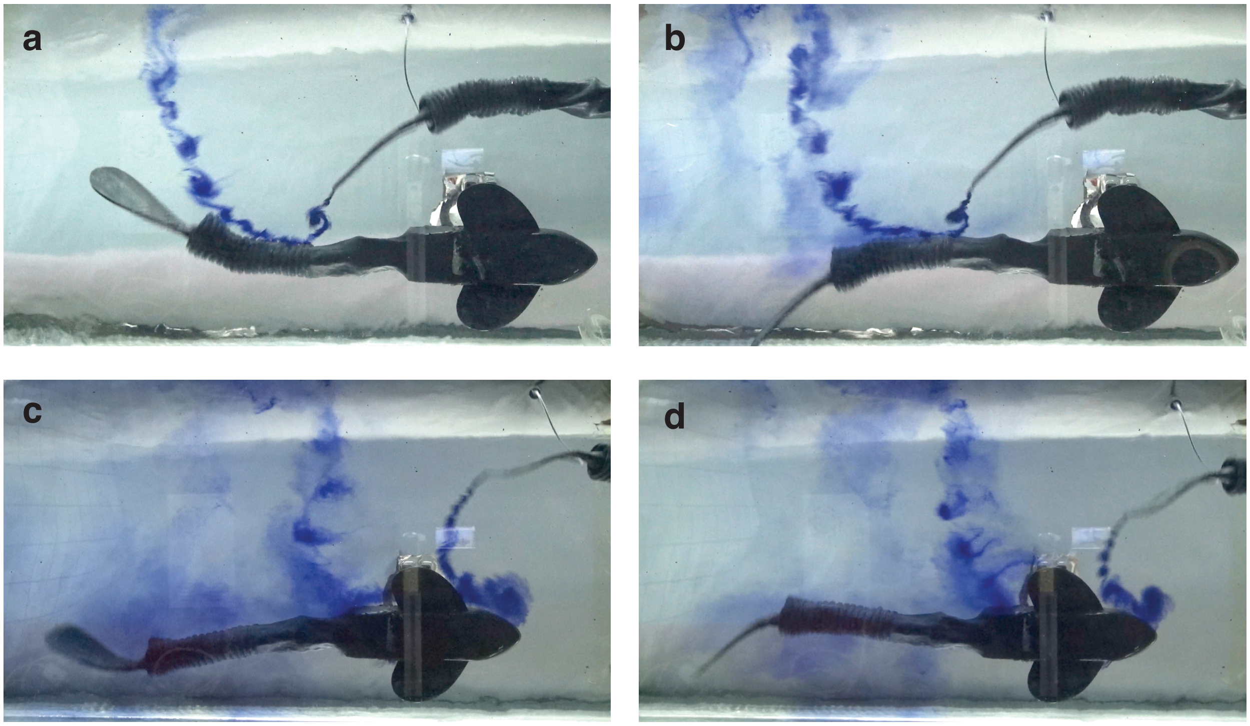

Supplementary Fig. 9: **Dye visualisation of the hydrodynamic interactions between two robots with different spatial relationships (bottom view)**. Robotic fish swim in a moderate (**a, b**) and large (**c, d**) front-back distance with in-phase (**a, c**) and out-phase (**b, d**). A strong hydrodynamic interaction between the vortices and the follower's body occurred when space difference is moderate (approximately 0.5 BL). The interaction almost vanished when the distance is as large as 1 BL.

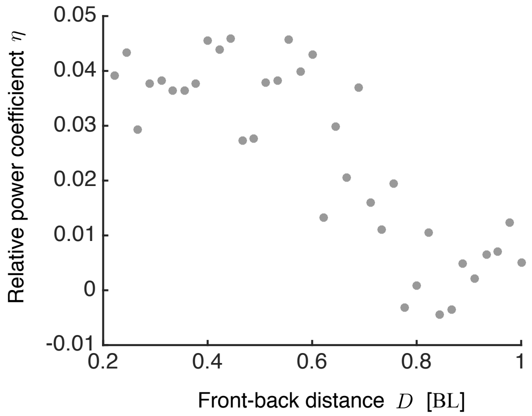

Supplementary Fig. 10: **Maximum relative power coefficient η as a function of the front-back distance D.** The values decay strongly as a function of distance, indicating the beneficial effects of hydrodynamic interactions becomes dominated by turbulence over distances greater than approximately 0.7 BL.

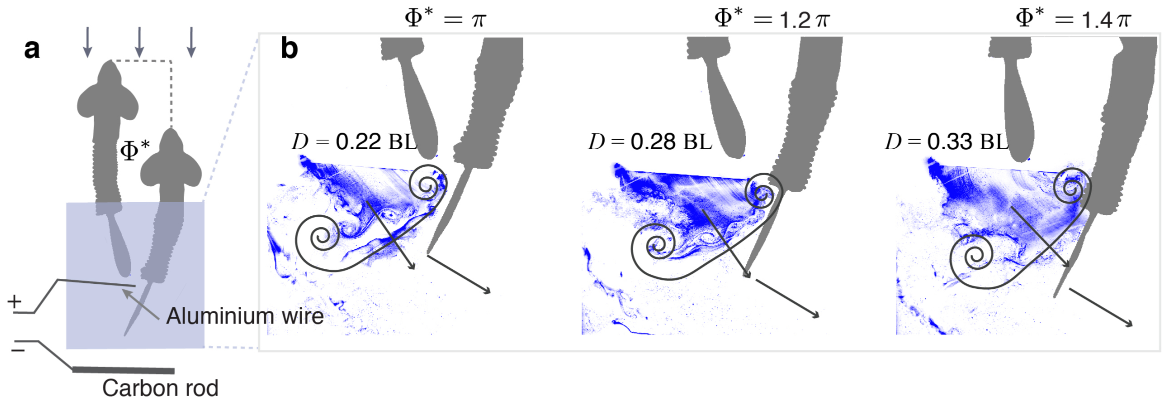

Supplementary Fig. 11: **Hydrogen bubble visualisation of the mechanism by which vortex phase matching results in energy savings.** (**a**) A schematic of flow visualisation using hydrogen bubbles. The shaded area is visualised using a high speed camera. (**b**) Visualisation of hydrodynamic interactions between two robots swimming at left-right distance G= 0.31 BL, front-back distance D= 0.27, 0.3, 0.33 BL and optimal phase differences $\Phi^* = \pi, 1.2\pi, 1.4\pi$. Among these energy-saving hydrodynamic interactions there are similar relationships between the vortex position and body phase of the follower. We call this relationship vortex phase matching (VPM).

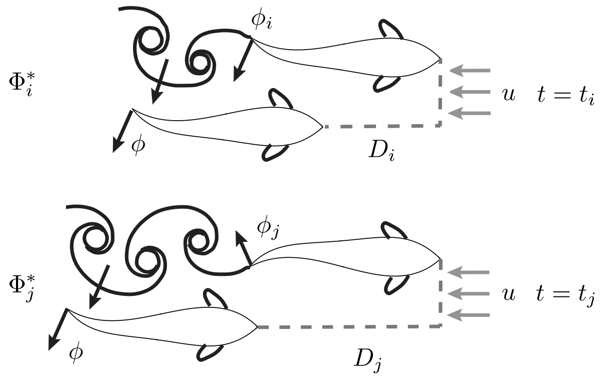

$$\Delta t = t_j - t_i = \frac{\Delta\Phi}{2\pi \cdot f} = \frac{\Delta D}{u}$$

Supplementary Fig. 12: **Illustration of the model derivation.** Under constant flow u, the follower saves most energy with phase difference Φ_i^* and front-back distance D_i at time t_i, and phase difference Φ_j^* and front-back distance D_j at time t_j. The time consumed for the vortex transfer from D_i to D_j equals the time taken for the leader to undulate its tail from body phase ϕ_i to body phase ϕ_j. During vortex phase matching (VPM) the follower interacts with the vortex shed by the leader at any given front-back distance with the respective phase. During a continuously changing front-back distance the phase difference changes also continuously. When the matching occurs, then the time needed for the vortex to travel ΔD distance in a flow that has a speed u is equal to the time of the phase changes accordingly defined by the frequency f of the undulation.

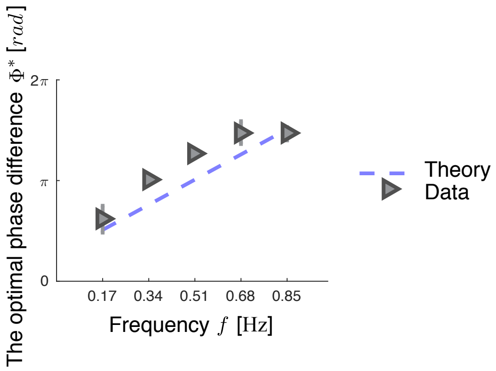

Supplementary Fig. 13: **A test of the vortex phase matching (VPM) rule with various frequencies at front-back distance D=0.31 body lengths (BL), left-right distance G=0.33 BL, flow speed u=0.054 body lengths per second (BLs^{-1}).** All the experiments are conducted with the robotic fish fixed in the flow tank. The optimal phase difference is the phase difference for which the energetic costs to the follower are minimised. Error bars are standard error of the mean.

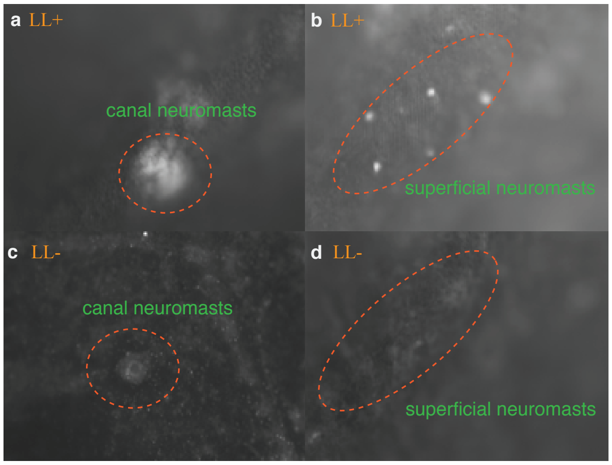

Supplementary Fig. 14: **Microscopic images of the intact and impaired lateral line.** Intact (**a, b**) and impaired (**c, d**) lateral line under the epi-fluorescence stereomicroscope for canal neuromasts (**a, c**) and superficial neuromasts (**b, d**).

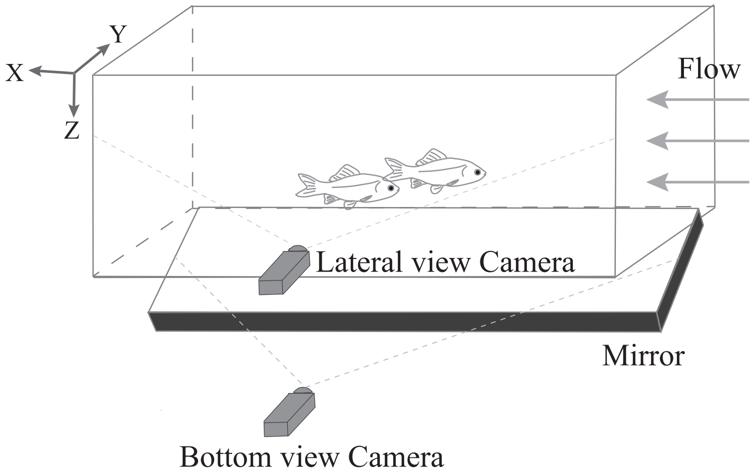

Supplementary Fig. 15: **A schematic showing the setup for the real fish experiments.** A mirror is placed at the bottom of the tank at a 45 degrees with respect to the horizontal plane. The lateral and bottom views are synchronised to record with same frequency.

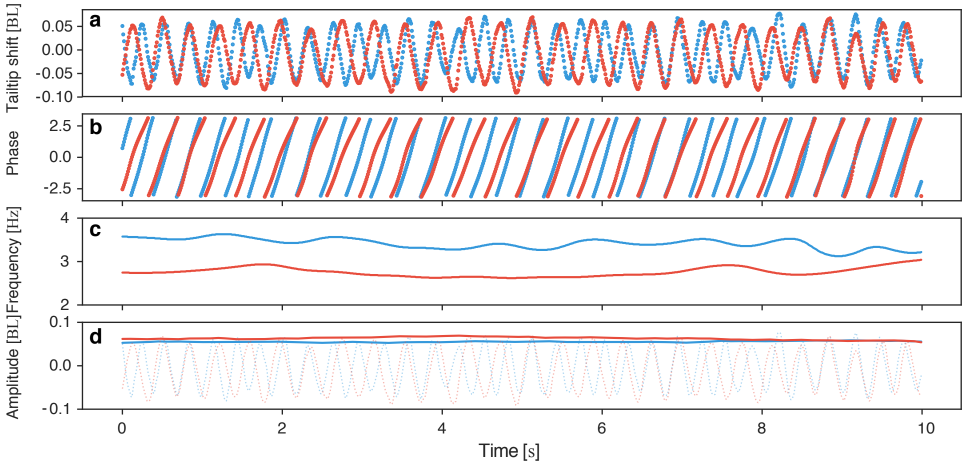

Supplementary Fig. 16: **Example of fish swimming characteristics.** (**a**) Lateral position of the tailtip (measured at caudal peduncle). (**b-d**) phase (**b**), frequency (**c**) and amplitude (**d**) of the corresponding fish movement.

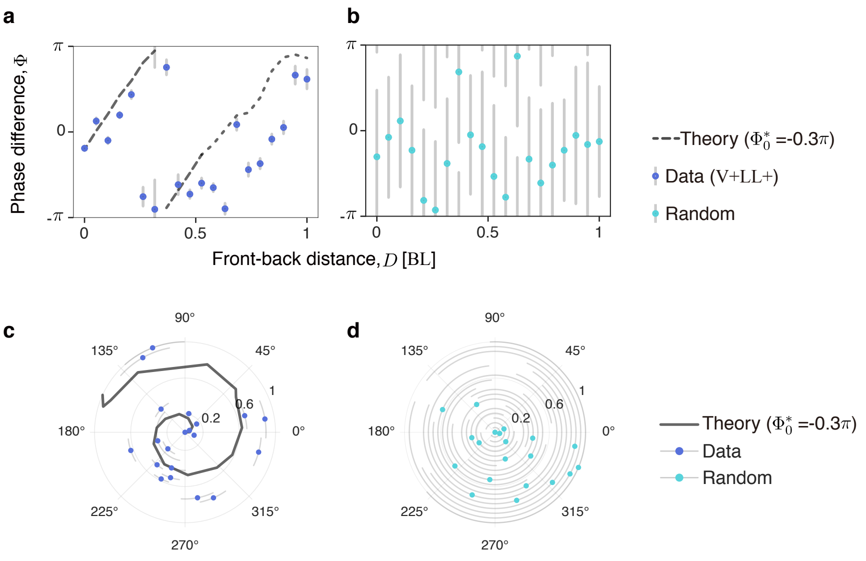

Supplementary Fig. 17: **A comparison between data and vortex phase matching theory, and the randomised data for the intact case (V+LL+, intact vision and intact lateral line).** Comparisons are shown in both Cartesian coordinates (**a**, **b**) and polar coordinates (**c**, **d**). For each bin (at D with a bin size 0.05 BL) of data, theory and the randomised set we calculated a circular mean and circular standard error by Von mises fitting after 1000 bootstraps (see supplementary methods). Error bar is shown periodically within $-\pi$ to π.

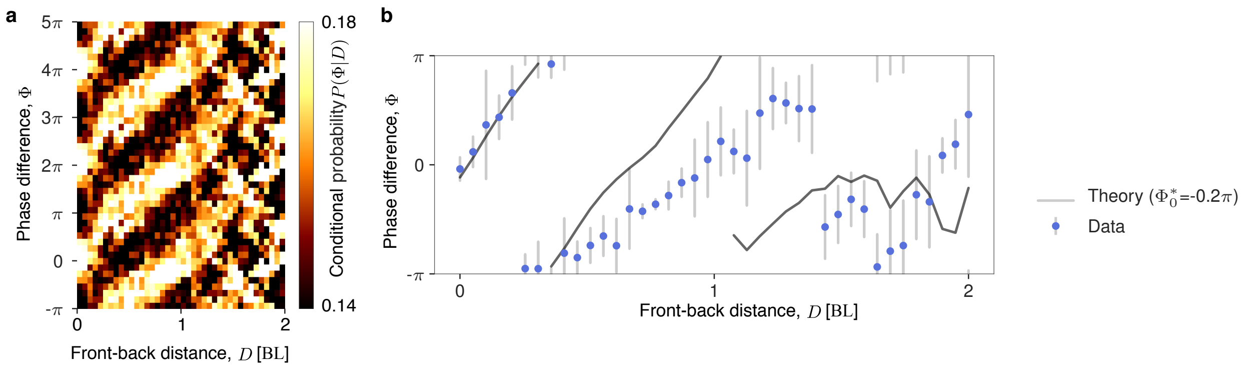

Supplementary Fig. 18: **Phase difference distribution versus front-back distance until 2 body lengths (BL).** (**a**) Phase difference distribution along the range of front-back distance. (**b**) The vortex phase matching (VPM) model fits data (circular mean and circular standard error) when the front-back distance is within 0.5 BL, where the hydrodynamic interactions are strongest. After the front-back distance is larger than 1 BL, there is still an anisotropy in the observed phase differences but the fitting to the theory starts to deviate significantly. The deviation could be due to vortex dissipation and turbulence, which are both not captured in the inviscid assumption presupposed in our theory. We note that the same phenomenon also occurs in the strictly controlled robotic fish system when the front-back distance is around 1 BL (see Fig. 2**a, b**).

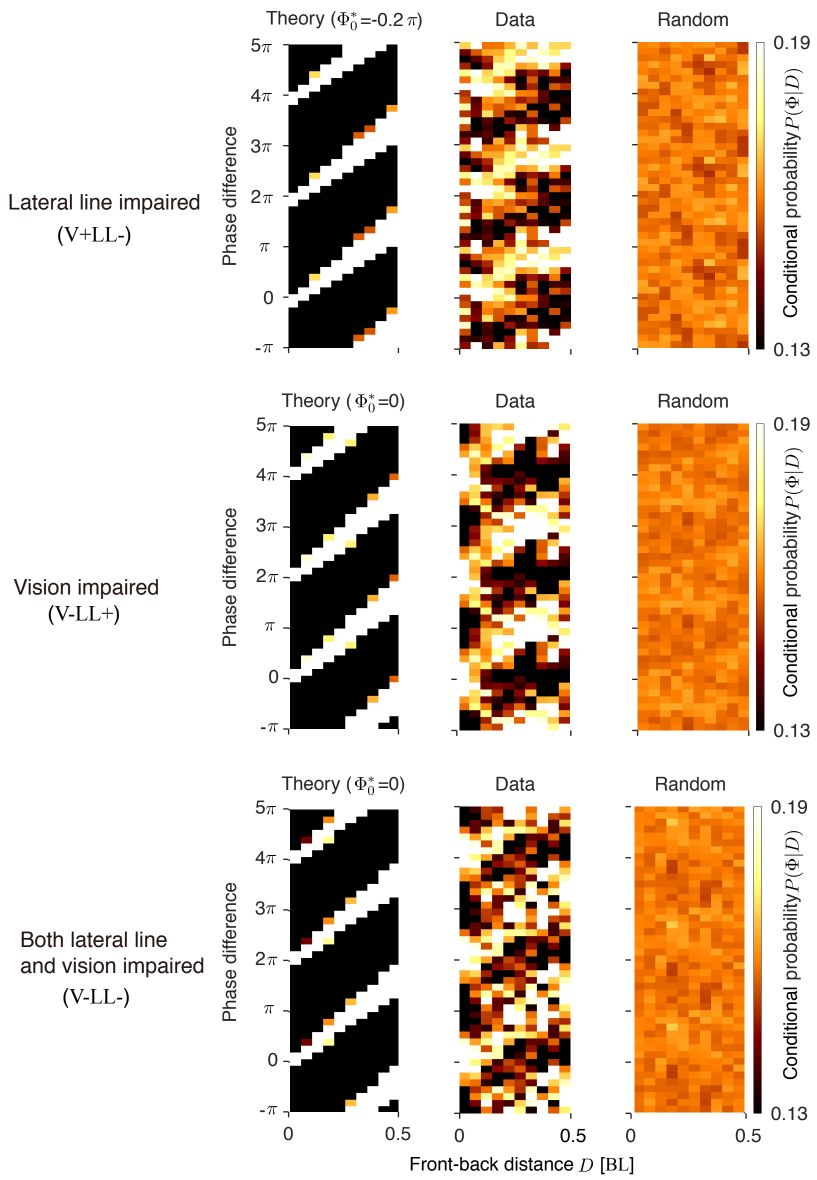

Supplementary Fig. 19: **Phase difference versus front-back distance when the sensory systems are impaired.** For the theory, we used Φ_0^* values fitted to the data (and shown above plots). The data in all cases show significantly similar behaviour to the theory as compared to the randomised set (V-LL+: $P < 0.04$, V+LL-: $P < 0.002$, V-LL-: $P < 0.006$). The theoretical predictions fit the data well up to approximately 0.5 BL and then diverge due to vortex dissipation and turbulence. See Supplementary Fig. 18 for more details.

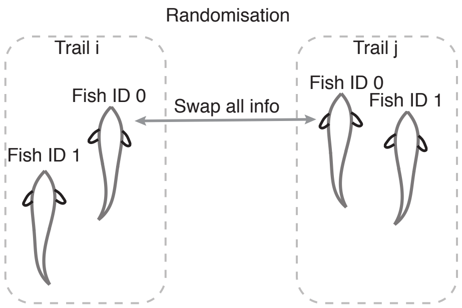

Supplementary Fig. 20: **Schematic explanation of the randomisation.** Randomisation is conducted by swapping all information (positions and body postures) of two fish respectively from two random trials within same treatment.

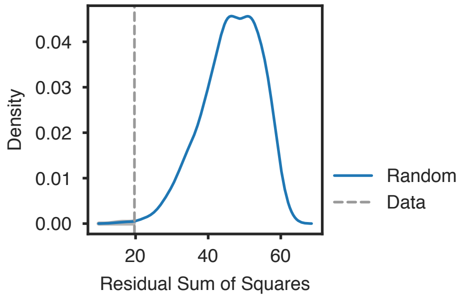

Supplementary Fig. 21: **Results of the randomisation test.** The distribution of the residual sum of square values given by the randomisation test (blue curve) as compared to the value observed in the real data (grey line). The distribution shows residual sum of square (RSS) circular mean fitting of the theory for all 1000 randomisations and the data. The vertical line indicates the value of RSS between the data and the theory. Shadow area (the grey area near the left tail of the distribution) shows when the randomisation gives equal or lower RSS compared to the the observed data. The proportion of the shadow area over the whole area of the distribution is 0.002 (0.2%).

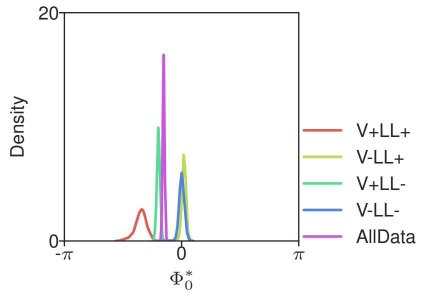

Supplementary Fig. 22: **Distributions of optimal initial phase difference Φ_0^* after 1000 bootstraps for all 4 treatments (intact: V+LL+ (intact vision and intact lateral line), three sensory impairments: V-LL+ (impaired vision and intact lateral line), V+LL- (intact vision and impaired lateral line) and V-LL- (impaired vision and impaired lateral line)).** All Φ_0^* located inside the energy saving zone shown in Fig. 4**a-c**

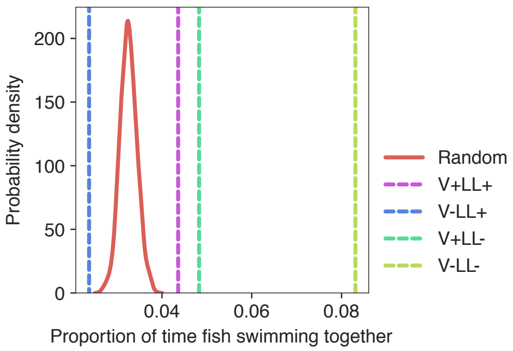

Supplementary Fig. 23: **The proportion of time fish swim in close proximity (front-back distance $D < 1$ body lengths (BL)) for each treatment group.** The distribution shows the proportion of values we obtain using 1000 randomisations. All of the values of the randomisation are lower than that observed in the real data (V+LL+). In only one treatment case (vision impaired: V-LL+) we find that fish swim less frequently together than what is expected by random chance. Fish in which both vision and the lateral line impaired (V-LL-) swim much more often together.

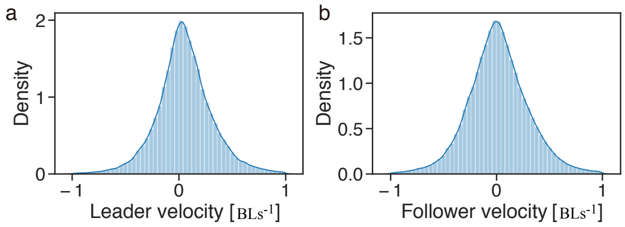

Supplementary Fig. 24: **The distribution of speed of leader and follower in the coordinate system of the flow tank.** The speed of both leader and follower is around 0, which indicates fish adopts the speed of the flow and they swim with relative stable formation during vortex phase matching.

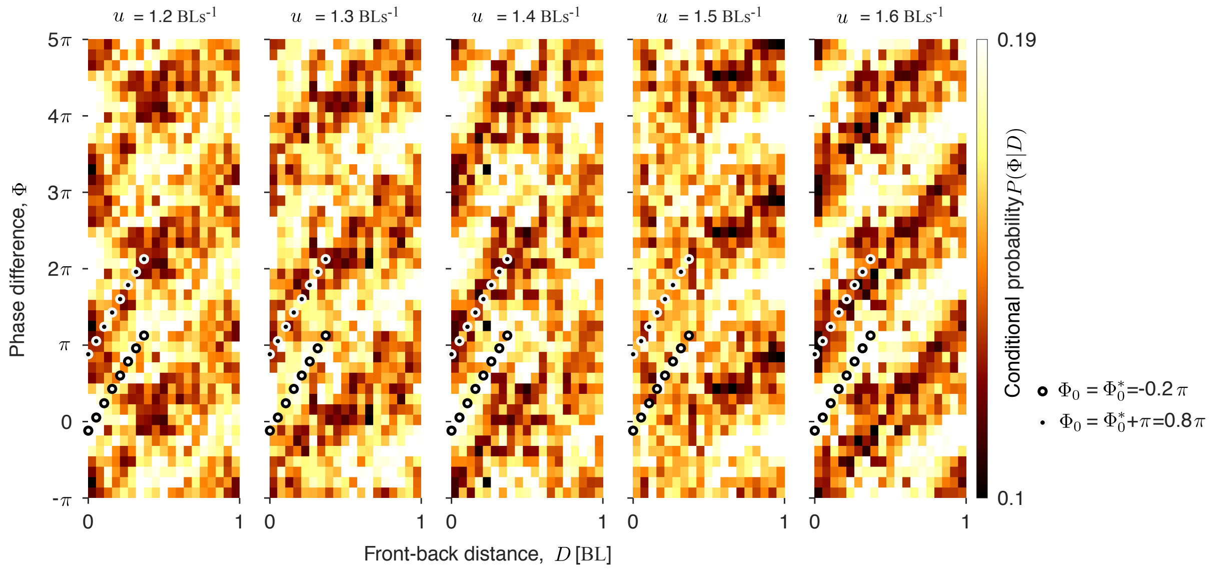

Supplementary Fig. 25: **Vortex phase matching and its relation to energy saving for different flow speed in real fish.** Data are pooled from all treatments. Plots show the phase difference distributions Φ (y-axis) versus front-back distance D (x-axis) at different flow speeds. White circles (with black outline) indicate the most frequently occurring phase differences (corresponding to Φ_0^*) for each bin within 0.4 BL front-back distance. Black circles (with white outline) mark the opposite phase as compared to Φ_0^*. In Fig. 4**a-c**, we show the amplitude A, lateral moving speed v_y, and frequency f during those instances that the fish fell within these regimes. More generally, we compared any Φ_0 to its opposite phase and in Fig 4**a-c**, $A - A^{+\pi}$ is calculated by $A(\Phi_0)$-$A(\Phi_0 + \pi)$. $v_y - v_y^{+\pi}$ is calculated by $v_y(\Phi_0)$-$v_y(\Phi_0 + \pi)$. $f - f^{+\pi}$ is calculated by $f(\Phi_0)$-$f(\Phi_0 + \pi)$.

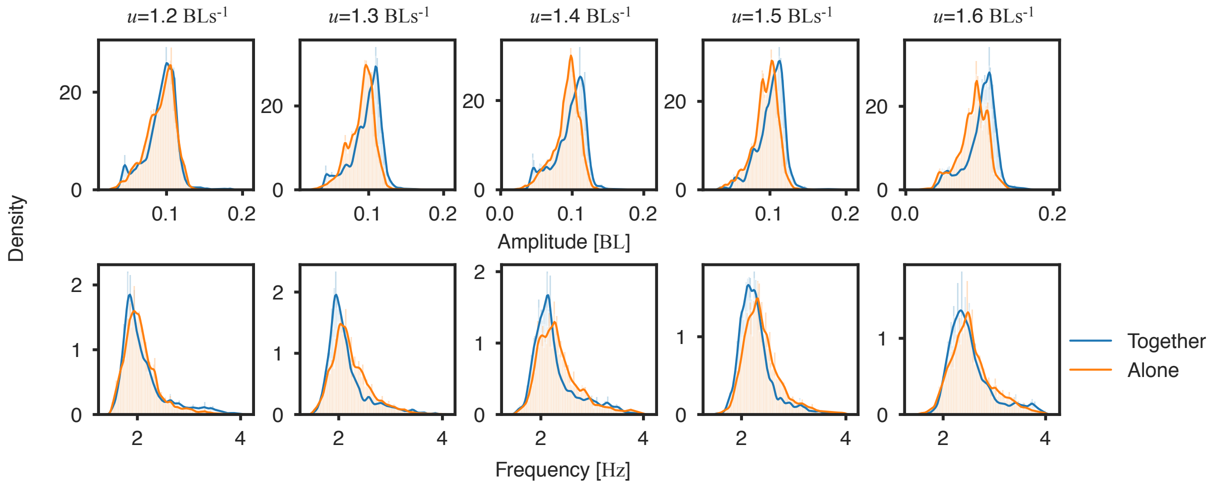

Supplementary Fig. 26: **Frequency and amplitude comparison when swimming together and alone.** We defined instances of swimming alone when the front-back distance D between the two fish was larger than the range of the possible hydrodynamic interactions ($D > 2$ BL). Schooling (swimming closely together) result in lower frequency but larger amplitude as compared to swimming alone.

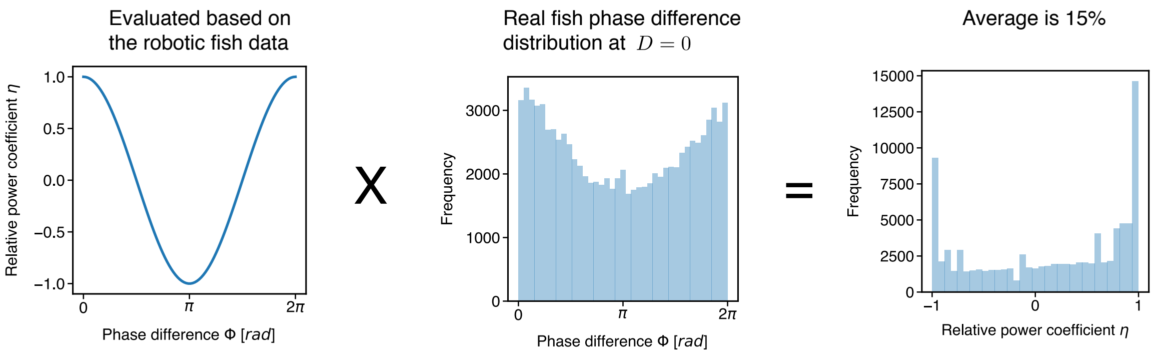

Supplementary Fig. 27: **Schematic to show how we evaluate the power cost of the real fish based on the robotic fish data.**

Supplementary Note 1

Robotic locomotion control

We employed a bio-inspired locomotion control algorithm (based on the Central Pattern Generator, CPG)[2,3] for the behaviour of the robotic fish. Formally, this model can be described as:

$$\dot{r}_i(t) = \alpha\ (R_i - r_i(t)) \tag{1}$$

$$\ddot{\phi}_i^c(t) = \sum_{j=1}^{3} \mu_c\ [\mu_c\ (\phi_j^c(t) - \phi_i^c(t) - \varphi_{ij}) - 2(\dot{\phi}_i^c(t) - 2\pi f_c)] \tag{2}$$

$$\theta_i(t) = r_i \cos(\phi_i^c(t)) \tag{3}$$

Where r_i, ϕ_i^c, θ_i are the state variables representing the undulation amplitude, phase and angle of the ith joint. R_i and f_c determine the intrinsic amplitude (in radians) and frequency (in Hz), respectively. φ_{ij} is the phase bias, which defines the coupling between oscillators controlling joints i and j. α and μ_c are system parameters which determine how fast the control responses to the input parameters. Both were set as 10 s^{-1} in our system, matching the dynamic response time of the servomotor. We fitted the remaining parameters of the controller be similar to the motion of free swimming fish (subcarangiform swimmers) and the resulting values were in agreement with a previous study on fish locomotion[1]. The values used were $R_1 = 0.0667$ rad, $R_2 = 0.1222$ rad, $R_3 = 0.1444$ rad, $f = 0.85$ Hz, $\varphi_{12} = 1.396$ rad, and $\varphi_{13} = 2.094$ rad. Namely, the robot swam with amplitude 0.1015 m, frequency 0.85 Hz,

free swimming speed 0.245 ms^{-1}. See reference [3] for a detailed analysis of this controller.

Parameter setting for the experiments with robotic fish

In order to determine the parameter region of interest for robotic fish where hydrodynamic interactions may be possible, we relied on previous literature[4,5] and our experimental approaches. Our flow tank was sufficiently wide to allow the range of left-right distance G that is typical of two real free-swimming fish[4,5] (range of 0.27 to 0.33 BL; with an increment of 0.022 BL). For the front-back distance, D, the smallest distance possible was approximately 0.22 BL, due to the physical properties of the pectoral fin of the robotic fish. In order to determine the maximum front-back distance, we relied on visualisation of the hydrodynamic interactions between the vortex shed by the leader (the front robotic fish) and the body of the follower (the rear robotic fish) (see Supplementary Fig. 9). A dye probe was attached on the centre of the tail of the leader and supplied with dye by a gravity feed. The interactions between the vortices and the body posture of the follower were recorded from the bottom view by a Sony camera (HDR-GWP88E, 100 fps). For moderate front-back distances (approximately 0.5 BL), we observed a strong hydrodynamic interaction between the vortex and the follower's body (see Supplementary Fig. 9**a, b**). When D reaches approximately 1 BL, the vortex phase interactions become vanishingly small due to vortex dissipation (see Supplementary Fig. 9**c, d**). Therefore, we set the maximum front-back distance D as approximately 1 BL.

Measurement of the power consumption of the robotic fish

Relying on simple electrical principles, we were able to measure the power consumption of each robot fish as the product of the voltage and the current. Given that our robotic fish are powered by a stabilised power supply, the voltage is effectively constant, and the current is directly proportional to represent the power costs. In order to verify the effectiveness of our approach to measuring power, we conducted a Fourier analysis of the measured power values (see Supplementary Fig. 4). The main frequency is 1.7 Hz which is double of the frequency of the body undulation of the robotic fish (0.85 Hz), consistent with the assumption that energy costs match the temporal characteristics of locomotion.

Measurement of the net force exerted on the robotic fish in the flow tank

To test the net force of the robot swimming in the flow tank, we connected the robotic fish to a load cell (HBM Z6FC3) to measure the net force in the front-back direction. As a reference, we first measured the force when the robot is static and at zero flow speed. We, subsequently, measured the net force when the robotic fish undulates (the kinematic parameters are the same as above) at different flow speeds. The net force as a function of the flow speed is shown in Supplementary Fig. 5. The flow speed (0.25 ms^{-1}) which results in a net force of around 0 (the net force of the drag and trust generated by body undulations) is similar to

the free swimming speed (0.245 ms^{-1}). This indicates that our setup with the robotic fish swimming in the flow tank represents reasonably the hydrodynamics of free swimming.

Visualisation of vortex interactions

In order to better understand the hydrodynamic interactions that determine energy savings, we visualised the flow in the wake of the robotic fish using a combination of dye injection and hydrogen bubbles. First, we examined the shed vortices from a freely-swimming robotic fish using dye injected by a thin needle attached to the centre of the caudal fin. We recorded the resultant vortices using a digital camera (Logitech c920) (Supplementary Movie 1). As this method is not suitable for visualising hydrodynamic interactions between robotic fish, for this we relied on a sheet of hydrogen bubbles and a laser light sheet (.2mm thick, 7W, Laserwave, Beijing, China). Hydrogen bubbles were generated by suspending an aluminum wire in front of the robot (as a cathode) and a sheet of metal behind the robot (as an anode) and running direct current (DC) to these two pieces of metal (Supplementary Fig. 11**a**). This produced a reliable sheet of bubbles which could be laser illuminated and recorded using a high speed camera (CMOS Histar 8G type) with 100 frames-per-second (Supplementary Fig. 11**b** and Movie 3). Although the presence of hydrogen bubbles could bias the exact hydrodynamical properties, we only used this method as a confirmation, and not as a quantitative analytical approach.

Supplementary note 2

In order to further test the effect of the bar in our platform, we conducted Computational Fluid Dynamics (CFD) simulations with two numerical robotic fish swimming stably side-by-side. We developed the CFD model based on HyperFLOW (Hybrid Platform for Engineering and Research of FLOWs) developed by China Aerodynamics Research and Development Center. The fish model is designed according to the 2D projection of the robotic fish body. Locomotion of the numerical robotic fish is the same as that applied in robotic fish. A strong coupling solver is applied to solve the coupled Navier-Stokes equations, rigid body dynamics (RBD) equations and the self-control of body deformation.

The simulation is conducted using an unsteady Navier-Stokes solver under the Arbitrary-Lagrange-Eulerian (ALE) framework:

$$\frac{\partial}{\partial t}\int_V U \mathrm{d}V + \oint_S (F_i(U) - U v_g \cdot \mathbf{n})\mathrm{d}S = \oint_S F_v(U)\mathrm{d}S \tag{4}$$

where S is the surface surrounding the control volume V, $\mathbf{n}$ is the out-going unit normal of S, v_g is the velocity of S, and U is the vector of conservative variables, F_i is the inviscid and F_v is the viscous flux vectors.

The semi-discrete form of the RBD is:

$$\frac{\partial Q}{\partial t} = F(Q, P) \tag{5}$$

where Q is a vector, representing the kinetic parameters, P denotes the fluid forces acting

on the fish body. The time marching step can be written as:

$$Q^{m+1} = (1-\varepsilon)Q^m + \varepsilon[aQ^n + bQ^{n-1} + \Delta t(cF^m + dF^n + eF^{n-1})] \tag{6}$$

where m and n are the index of inner and outer iteration respectively. $\varepsilon = 0.1$ is the relax coefficient. Different values of the parameters a, b, c, d, and e will give different temporal accuracy. Here, we adopted the 2nd-implicit Euler temporal scheme with $a = \frac{4}{3}$, $b = -\frac{1}{3}$, $c = \frac{2}{3}$, $d = 0$ and $e = 0$. More details can be found in reference [6].

The kinematics of the numerical robotic fish is same as the robotic fish (see Supplementary Equations 1-3).

The power costs of swimming P_{Total} are evaluated by:

$$P_{\text{Total}} = -\iint \mathbf{u} \cdot \mathrm{d}\mathbf{F}, \tag{7}$$

where $\mathbf{u}_{\text{Def}}$ represents the deformation-velocity of the fish's body.

Details of this CFD model are described in our previous work[6]. Power costs of the robots were measured and compared at different phase differences (Supplementary Fig. 6**b, d**).

Supplementary note 3

We consider a deliberately-simplistic hydrodynamic model in which, for a pair of fish (a leader and a follower), the follower attempts, depending on its relative spatial position with respect to the leader, to optimise the phase of its tail undulations with respect to that of the leader, in order to minimise energetic expenditure, or to maximise thrust (Supplementary Fig. 12). We assume the fish rely solely on vortex phase matching (VPM, described in the main text) and that the flow is inviscid. In accordance with vortex phase matching, fish can adopt some optimal body phase ϕ to interact with the vortex present a given time t_i and distance D_i. As the vortices drift downstream, that same optimal body phase ϕ occupies a new position in space D_j and time t_j. Since the flow is assumed to be inviscid, the spatiotemporal distance between these points can be trivially characterised in accordance with the speed at which the vortices move away, in the direction of the flow, from the leader, u. As a consequence, the distance between these locations is equal to the speed of the vortices multiplied by the time elapsed, $\Delta D = u\Delta t$. In other words:

$$\Delta t = t_j - t_i = \frac{\Delta D}{u} \tag{8}$$

At t_j, we wish to determine how the optimal phase difference between the leader and follower has changed (*e.g.* $\Phi_j^* - \Phi_i^*$). As the vortex phase matching strategy assumes (above) the follower's phase (ϕ) would be identical between these two spatiotemporal points, this difference depends only on the change in the leader's phase ($\phi_j - \phi_i$). We wish to characterise

how the phase difference changes between these points ($\Delta\Phi$), and can do so as follows:

$$\Delta\Phi = \Phi_j^* - \Phi_i^* = (\phi_j - \phi) - (\phi_i - \phi) = \phi_j - \phi_i \tag{9}$$

Given that a fish would need one period to advance 2π phase units, the time it will take for the lead fish to advance from ϕ_i to ϕ_j is:

$$\Delta t = \frac{\phi_j - \phi_i}{2\pi} \cdot T = \frac{\Delta\Phi}{2\pi f} \tag{10}$$

According to Equation 8 and 10, time consumed for the distance changing and phase changing equals:

$$\frac{\Delta D}{u} = \frac{\Delta\Phi}{2\pi f} \tag{11}$$

Which can be rewritten as:

$$\Delta\Phi = \frac{2\pi f}{u}\Delta D \tag{12}$$

We further define Φ at D=0 as Φ_0, which describes how the follower reacts to the vortex when front-back distance is 0. Consequently, Equation 2 (in the main text) describes the relationship between the phase difference Φ, and the front-back distance D. The exact assumptions and their possible consequences are discussed in the following discussion section, but the validity of these assumptions is indirectly provided by the good match between the theoretical predictions and the experimental data.

Supplementary Note 4

Real fish

32 juvenile goldfish (*Carassius auratus*), (20±4 *cm*) were obtained from a commercial supplier in Germany (www.fischfarm-schubert.de). We kept fish in a temperature controlled room at approximately 12 ^oC, with 12:12 day:night light cycle, for 1∼2 weeks before the start of the experiments in two tanks containing 6 fish each. Each fish was fed everyday in the morning at around 9:00AM with food equivalent to 10% of the weight of each fish's body. 12 individuals were randomly picked and tested under mixed colour, visible spectrum (white) LED light with intact lateral line (V+LL+), 10 individuals were tested under only inferred light (850 nm) with intact lateral line (V-LL+), 6 were tested under visible spectrum LED light with lateral line inhibited (V+LL-), and 4 were tested under inferred light with lateral line inhibited (V-LL-). Experiments were conducted during 10/12/2017∼21/12/2017 and 23/07/2018∼27/08/2018. All animal handling and experimental procedures were approved by Regierungspräsidium Freiburg, 35-9185.81/G-17/90.

Lateral line impairment

For the lateral line impairment, we followed the procedure described in reference [7]. Specifically fish were immersed in a 6 Litre treatment of 2.7 g neomycin for 3 hours. Subsequently,

we conducted experiments following the protocol of the intact fish. To ensure that the dosing employed was sufficient, after the experiments, we randomly chose one fish from the pair fish and put it in 0.006% DASPEI (2-[4-(Dimethylamino)styryl]-1-ethylpyridinium) solution for 10 minutes. Following this, the fish was anaesthetized by immersion in 0.15 g/L MS-222. We then observed the surface of the fish under an epi-fluorescence stereomicroscope (Apo-Tome.2) to determine whether fluorescent DASPEI dye had been taken up by the lateral line hair cells[7]. Since failure to uptake DASPEI indicates non-functioning transduction channels, a lack of visible flouresence was indicative of lateral line impairment. This was in stark contrast to a randomly selected, un-treated fish where, as found in numerous previous studies (Ref. [7, 8] and citations therein) the lateral line cells are highly conspicuous. A comparison is shown in Supplementary Fig. 14.

Data collection

Based on the posture tracking results, we calculated phase ϕ and amplitude A using a Hilbert transformation. To calculate instantaneous swimming frequency we applied a time-frequency transform to the tail tip movement data using normalised Morlet wavelet convolution[9]. We used 1000 dyadically-spaced frequencies from 0.5 Hz to 10 Hz with a non-dimensional parameter of 5 ($\omega_0 = 5$). We selected peak frequency based on the maximum amplitude of the frequency spectrum at each time point. We then filtered the complete dataset to extract a subset wherein the follower is likely to be interacting with the vortex shed by the leader,

according to the following criteria:

1. Front-back distance D ranges from 0 to 1 BL.
2. Left-right distance G ranges from 0.1 to 0.33 BL.
3. Tail-beat frequency f ranges from 1.5 Hz to 6 Hz.
4. The height different between the fish is within 0.5 of the body height.

Data processing

After the filtering, we were left with a subset that included front-back distance D, left-right distance G, phase ϕ, frequency f, and amplitude A.

Data: With these values, we calculated the phase difference between the leader and the follower as:

$$\Phi = \phi_{\text{leader}} - \phi_{\text{follower}} \tag{13}$$

Theory: Our hypothesis, resulting from our robotic experiments and hydrodynamic theory, is that grouping fish may be utilising vortex phase matching (VPM) in order to minimise costs of locomotion, and thus we evaluate phase difference predictions by setting $\Phi_0 = 0$

according to our theory:

$$\Phi = \frac{2\pi f}{u} D + \Phi_0 \tag{14}$$

Theoretical values are given by setting $\Phi_0 = \Phi_0^*$ which is determined by fitting a mean value to the measured phase difference from the data. Details can be found in the following descriptions.

Randomisation: For comparison, we generated yoked control data using the same criteria after randomly swapping the full track (position and body posture) of two fish respectively from two randomly chosen trials (see Supplementary Fig. 20). Overall, we conducted 1000 randomisations for comparison with the experimental data.

Distribution plots: We then quantified (for actual and randomised data) the proportion of time following fish spent adopting an undulation with a given different phase difference ($\Phi = \phi_{\text{leader}} - \phi_{\text{follower}}$) as a function of front-back distance D. We used this method over all data within a given range of front-back distances (0.05 BL as a bin). These data were then normalised across bins to generate an overall probability distribution. This allowed us to estimate the most likely phase difference for a given position. For example, in Fig. 3**d** fish are most likely to swim with a phase difference Φ around 0 at front-back distance D=0.

Bootstrap: To characterise the accuracy of these measures, we used a simple resampling-type bootstrap method: we randomly sampled distributions of phase difference (with replacement,

using a Mersenne twister random number generator) from the original distributions of phase difference for each front-back distance (bin), with the same total number of data points as the original ones. These bootstrap samples have similar properties to the original histogram, so we recorded the locations of their maxim, for 1000 samples for each bin.

Von Mises fitting: Since phase difference Φ is an example of circular data, we fit Φ values with Von Mises distribution for each certain front-back distance (bin)[10]. These distributions are characterised by:

$$f(x|\mu,\kappa) = \frac{e^{\cos(x-\mu)}}{2\pi I_0(\kappa)} \tag{15}$$

where μ is a measure of central tendency, κ is the reciprocal measure of dispersion (κ is analogous to $1/\sigma^2$). Finally, $I_0(\kappa)$ is the modified Bessel function of order 0. We further applied a bootstrapping method on the distribution of the phase difference values for each front-back distance (bin) and then conducted Von Mises distribution fitting to obtain the value that represents the highest peak μ. Circular mean and circular standard error were applied to all the μ after bootstraps (see Supplementary Fig. 17). We performed 1000 bootstraps for experimental, theoretical and randomised data.

Periodic least square fitting: To evaluate Φ_0^*, we calculated periodic least squares differences for average μ of data by taking average theoretical μ as the fitting function and Φ_0 as the parameter. The algorithm shifts Φ_0 from $-\pi$ to π to find the Φ_0^* is associated with the minimum residual sum of squares. We further conducted Φ_0^* fitting for all 1000 randomisations.

A comparison of the residual sum of squares between data and 1000 randomisations is shown in Supplementary Fig. 21. The probability of obtaining the residual sum of squares of the data by random is very low (P=0.002, randomisation test see below for details).

Randomisation test between intact and three impaired cases: To quantify whether the measured distribution from each case (intact and impaired conditions) are more similar to each other as compared to a null expectation where the similarity would only appear by chance, we performed another renomination test. We first calculated the deviation between intact and each impaired case. We used the square-root of the squared differences for every bin as a single metric to characterises the deviation between the two distributions. We then applied the same procedure on the randomised data set, to obtain a distribution of deviation values which would appear by chance. We then compared the measured deviation to this distribution. In all pairwise comparisons, the intact and an impaired distributions were highly similar to each other and significantly different from the randomised cases, specifically we calculated the square root of the squared circular differences, Δ: V+LL+ vs. V-LL+: $P < 0.003$ (Δ_{data}=1.17, Δ_{random}=1.78 $\pm$ 0.23; mean $\pm$ SD), V+LL+ vs. V+LL-: $P < 0.001$ (Δ_{data}=1.05, Δ_{random}=1.8 $\pm$ 0.22), V+LL+ vs. V-LL-: $P < 0.001$ (Δ_{data}=1.13, Δ_{random}=1.8 $\pm$ 0.2). Therefore, this confirmed that the behaviour of fish regarding interaction with the phase of the vortex in both the intact and the impaired cases are similar to each other and thus, we can pool all data and analyse that together to obtain more precise statistics.

Swimming speed evaluation: We evaluated swimming speed in both stream-wise (front-back) direction v_x and lateral direction v_y. Distributions of v_x for both leader and follower are shown in Supplementary Fig. 24. The peak of v_x for both leader and follower is around 0, which indicates fish mainly swim stably during the hydrodynamic interactions.

Phase difference distribution within 2 BL front-back distance: We also verify our theory by comparing phase difference Φ distribution between strong and weak hydrodynamic interactions. For example, within 1 BL, the hydrodynamic interactions become stronger and their strength will increase as D approaches 0. And it will be weaker when distance is larger than 1 BL. Supplementary Fig. 18 shows Φ distribution within 2 BL, from all data. We can see that the linear correlation becomes weaker when the distance increases, especially when D is larger than 1.2 BL.

Phase difference when swimming side-by-side: We explored the phase difference when two fish swim side-by-side by adding a front-back distance filter ($D < 0.1$ BL). We performed 1000 bootstraps to obtain the phase difference by Von Mises fitting. The distribution of μ at D <0.1 BL is shown in Supplementary Fig. 22. The phase differences in all four cases are around 0 when the distance is within 0.1 BL. This indicates that the follower's tail moves in the same direction of the induced flow of the vortices shed by the leader, and thus that the follower kinematics are as expected for energy saving.

Vortex phase matching for energy saving: To explore why fish do VPM, we further analysed an estimate of the power consumption of the follower according to frequency f, amplitude A and lateral speed v_y. We pooled all data since fish do VPM for all four treatments (V+LL+: n=6, V+LL-: n=5, V-LL+: n=3, V-LL: n=2). Since high swimming speed will result to high swimming frequency, we analysed power consumption by dividing data according to the different flow speeds we used (see Supplementary Fig. 25). We mainly considered power consumption data at $D < 0.4$ BL because the hydrodynamic interactions are expected to be stronger when the follower is closer to the leader, and the further away the data contains more noise. The pattern in this regime has a clear sinusoidal behaviour.

Based on our robotic fish study (see Fig. 2), we know that moving the tail in the same direction as the induced flow of the vortices results in maximal energy saving; moving the tail in the opposite direction results in maximal energy costs. Other hydrodynamic interactions result in power consumption between these values. Moreover, there is a sinusoidal correlation between the power consumption of the follower and the phase difference (Fig. 4**a-c**). If such a sinusoidal correlation also exists in our data, and fish tail moves in the same direction as the induced flow to save energy, then this provides indirect evidence that fish employ VPM for saving energy.

Since Φ_0 determines the nature of hydrodynamic interactions, we conducted these analyses by shifting Φ_0 from $-\pi$ to π. To remove the effects of different flow speeds, we re-organised the frequency f, lateral speed v_y and amplitude A by subtracting values at Φ_0 by values at

$\Phi_0 + \pi$ (see Supplementary Fig. 25). Results are shown in Fig. 4**a-c** in the main text.

Φ_0^* determination: We also performed 1000 bootstraps to evaluate Φ_0^* for all four treatments (V+LL+, V-LL+, V+LL- and V-LL-). For each bootstrap, we conducted periodic least square fitting to obtain Φ_0^*. The distribution of Φ_0^* is shown in Supplementary Fig. 22. All the Φ_0^* are locating well within the energy saving zone.

Proportion of time fish spend by swimming together (and thus possibly performing hydrodynamic interactions): We show the proportion of time fish swimming together over all the videos for each treatment (V+LL+, V-LL+, V+LL-, V-LL-). The ratio is defined as:

$$P_{\text{data}} = \frac{\text{Number of frames fish are swimming together}}{\text{Number of all frames}} \tag{16}$$

Comparing to swimming alone: Frequency and amplitude are compared to fish swimming alone. We calculated frequency and amplitude of the leader as swimming alone when the front-back distance is larger than 2 BL. The frequency of follower shows that fish save energy comparing to swimming alone (see Supplementary Fig. 26)

Statistics

For each bin along the front-back distance, we employed Von Mises distribution fitting and 1000 bootstraps for intact (V+LL+) random and theory. Mean value and error-bar are

evaluated according to Von Mises fitting by circular mean and circular standard error[10]. Residual sum of squares are collected and compared between 1000 randomisations and data ($P < 0.002$, Supplementary Fig. 21). The similarity of the relationship between phase difference and front-back distance among all the four cases (V+LL+, V-LL+, V+LL- and V-LL-) are tested by comparing to 1000 randomisations (V+LL+ vs V-LL+: $P < 0.003$, V+LL+ vs V+LL-: $P < 0.001$, V+LL+ vs V-LL-: $P < 0.001$).

Supplementary Discussion

Why robotic fish

Using real fish to explore possible hydrodynamic interactions is challenging because there exist psychological effects, such as stress, which can also lead to higher energy cost than when swimming alone[11,12]. Furthermore, the changes in spatiotemporal relationships among fish may relate both to hydrodynamics and also other factors, such as moving to acquire information (such as about external features in the environment, or to obtain other types of social information). Therefore, it is very difficult to determine precisely spatiotemporal rules from tracking freely-swimming fish.

Most studies on energy saving mechanisms in fish schools have focused on understanding the possible interaction based on models based on principles and law of physics. These models can be divided into two main types: one is the numerical approach and the other is the empirical approach.

Due to advances in computational power, the numerical approach has been widely applied to study possible energy saving mechanisms in fish schools in the past decades. However, most still focus on 2D and low Reynolds number[13–15]. This is in stark contrast what can be observed in real systems which are characterised by occurring within a 3D environment and at high Reynolds number[16]. Moreover, most studies have estimated the power cost of fish

swimming based on the swimming speed and thrust/drag. This is inaccurate because drag and thrust are coupled while the tail is moving[17]. Therefore, obtaining a general rule based only on inherently in accurate numerical methods has proved possible but difficult[16].

Empirical models can be divided into passive empirical models and active empirical models. For the passive model, the typical approach used is to observe filaments of thin nylon wires in a soap flow tank[18,19]. However, this is different from the self-driven locomotion in real fish[13,20]. Active flapping empirical models mostly employ rigid foils[20–22] which may not approximate well fish body shape and their flexible caudal fin *etc.* (the later is thought to be important for the improvement of swimming efficiency[23]). Moreover, these physical models derive the energy cost of each individual *via* other measurable variables, such as evaluating the energy cost by the Euler-Bernoulli beam model[18], or by measuring the reaction torque on the motor and the angular velocity[21,24]. This is very challenging and complicated because the thrust and drag are coupled in a swimming system[25].

As a complement to physical models of filaments[19] and rigid foils[21,24], employing bio-mimetic robotic fish (see Fig. 1, Supplementary Fig. 1 and Movie 1) allows for similar body undulation, and thus generation of vortices similar to those created by real fish[13,26,27]. Such a bio-mimetic model also allows us to measure directly the power consumption of swimming by employing an ammeter with a stable power supply. This helps us to explore the relationship between power cost and the vortex phase interactions, both directly and conveniently. Moreover, since the robot can also swim freely, the flow speed is easy to set

according to the free swim speed.

The energy saving mechanism

With respect to the former hypothesis, initiated by the pioneering work of Weihs[28], several studies have suggested that fish should adopt a diamond formation[14,29–32]. More recently, diagonal formations have also been proposed as a mechanism for followers to save energy[15,30]. Other studies have proposed that fish could save energy by adopting a tandem formation in which one individual is directly ahead of another, at a specific distance[13,14,18,24,30]. Such a configuration has been studied by observing nylon filaments undulating in a soap flow tank[18], in computational fluid dynamic simulations[13,14,30], and in experiments with flapping rigid foils[24]. These studies suggest that followers may be able to extract energy from vortices, and thus benefit, if they position themselves at a specific distance behind the leader[13,14,18,20]. Several studies[19,24,33] even suggest that the leader may benefit if the follower is positioned closely to the leader. Further studies have suggested that rectangular formations[14,16], phalanx formations (side-by-side)[14] or triangular formations (two leaders swimming side-by-side and one fish swims behind)[30] may allow fish to save energy. Recent experiments employing simplified mechanical models[20,22,34], as well as theoretical studies[13,32], have also found that active swimmers could passively converge to specific stable spatial formations to save energy as a result of hydrodynamic interactions. We note, however, that experiments with real fish find that individuals do not adopt any such specific spatial configurations[35–37].

A non-mutually exclusive possibility is that a fish may adjust the phase relationship of its tailbeats, with respect to that of near-neighbours, in order to exploit neighbour-generated hydrodynamic forces[20,21,32,33,38]. For example, simulations of[38], and experiments employing[21], two foils flapping side-by-side find that being in phase maximally saves energy. Maertens *et al.*[33] simulated fish swimming with different tandem formations (only considering front-back distances, not lateral distances) and various phase differences, and their results suggest that phase difference is the primary factor allowing the follower to increase efficiency by saving energy, or to enhance thrust, with the effect of front-back distance being weak.

It is, of course, possible that both relative spatial position and the relative tailbeat phase, are important. Conducting experiments with two rigid foils swimming in-line, Boschitsch *et al.*[24] found that the thrust and propulsion efficiency of the follower is closely-related to the front-back distance and the phase difference with respect to the leader. In addition, in both 2D and 3D computational fluid simulations, deep reinforcement learning has been employed to predict where and how fish should place themselves in order to save energy[39]. These simulations suggest that follower fish should smartly[39] place themselves behind the leader where the relative spatial position and the phase difference are coupled by deforming their bodies to synchronise with the momentum of oncoming vortices—thus enhancing their swimming efficiency at no cost to the leader(s). If such a strategy is adopted by real fish, or how it may be achieved behaviourally, however, remains unknown.

The mechanism of energy saving found in our robotic fish experiments, termed vortex

phase matching (VPM), is determined based on both spatial and temporal variables. The follower needs to adjust its body undulation pattern according to the spatial positioning relative to the leader. This indicates that the energy saving is a dynamic process[39,40] and need not rely on individuals adopting constant spatial formations with respect to neighbours[14,28] (having little guidance as to what to look for in experimental data may have been one of the reasons as to why it has been very hard to find strong evidence for the energy saving hypothesis in real fish[35]).

As summeried above, previous mechanisms regarding energy saving resulting from schooling can be divided into two classes. One is spatial-based, which means the specific spatial formation among neighbours is the key to save energy. The other is temporal-based, which indicates the undulation pattern of the fish is most important. VPM is more general. If the temporal difference is constant, the mechanism of energy saving will converge to specific spatial formations. And the formation is determined based on the fixed value of the phase difference. This is consistent with previous studies[28,41]. If the spatial distance is fixed, the temporal difference will converge to relatively stable phase difference[38,40]. In reality, fish dynamically change their body phase according to the changing spatial difference. Previously, it was observed only that fish may be able to save energy in various formations but without understanding the underlying mechanism behind[37]. Our current theoretical and experimental work resolves these seemingly contradicting results (only temporal vs. only spatial coupling) by discovering a general spatiotemporal coupling described by vortex phase matching.

We also derive a simple analytical model that accurately describes the relationship between the optimal phase difference Φ^* and spatial difference D within changing of G. Although the derivation is based on the VPM (and the strong simplifying assumption of inviscid laminar flow; see supplementary methods and Supplementary Fig. 12), it fits both our robotic fish and real fish data very well (especially when the distance is small (D <0.5 BL; see Fig. 2, 4 in the main text)). Unsurprisingly the deviation for real fish, which might come from other hydrodynamic effects, such as the channelling effect[16,28] and the suction effect induced by low-pressure regions[42], is larger than that of artificial system. This is reasonable as real fish, as discussed above, are also responding to other factors in their environment, and in addition are subject to a wide range of random factors such as sensory noise and errors in sensory-motor transformation[43]. However, despite this variability we find strong effects of hydrodynamic interactions and this strategy is also very promising for applications in the field of engineering.

Spatial phase and initial phase difference

In our paper we used initial phase difference Φ_0 to describe the phase difference between the body and vortex shed by the leader (See Fig. 2**c**-**e**). Spatial phase and Φ_0 capture a similar concept, but for the Lagrangian and the Eulerian specifications of the flow field, respectively. We can describe the vortex-body interactions in either way, as both describe the same phenomena just using different coordinate systems.

If we track the wake and the body continuously and describe the path drawn by the tailtip (a sinusoidal track), it is better to describe in Lagrangian specification with spatial phase. If we are interested in the phase at a specific distance, however, it may be more intuitive to describe our analysis in the Eulerian specification with Φ_0, the phase difference at front-back distance 0. In our case, since the concept of Eulerian specification is also applied in the following real fish data analysis, we adopted the initial phase difference throughout the paper.

The initial phase difference Φ_0 and typical initial phase difference Φ_0^*

In our theory, parameter Φ_0 describes a fixed phase shift to allow for different types of interaction with the vortex. For example, if Φ_0=0 denotes that the follower moves its tail in the same direction as the induced flow of the vortices, then Φ_0=π denotes moving the tail in the opposite direction.

We include this parameter for the following reasons:

- The real fish may interact with the different phases of the vortex, as for example to achieve the highest thrust possible, or to save energy. Namely, fish interacting with vortices may do so not exclusively for saving energy.

- The phase of the vortex is calculated from the phase of the leader fish. When and where the vortex is being formed could introduce a constant phase shift in a real life system. We note that the differences between the robotic and real fish anatomy, and the way the experiment was performed (robots are connected to the six-axis platform respectively with a thin bar, while the real fish are free swimming), does not necessarily allow a precise comparison comparison of the Φ_0 values between the robotic and real fish experiment.

- In our model we assume that energy savings result from the hydrodynamic interactions between the fish body and the induced flow of the vortices shed by the leader. Without loss of generality, we simplify this as an effective point of the fish body that interacts with the flow. Based on this points phase we can define the relevant phase relationship between the leader and the follower, thus we get the initial phase difference Φ_0^*. However, this effective point varies from system to system due to many factors such as fish body morphology, body kinematics and body friction. In the real fish data analysis we tracked the caudal peduncle since this represents well the body phase of the fish (Supplementary Movie 5). However this point may be slightly shifted from the effective point, resulting in a shift of the body phase. Therefore, the constant initial phase differences Φ_0^* may differ between systems, such as between our robotic and real fish.

From our further power consumption analyses, we know $\Phi_0 = \Phi_0^*$ results in energy saving

hydrodynamic interactions. Therefore, in our data analyses, $\Phi_0 = \Phi_0^*$ represents the regime in which energy saving is maximised.

Hydrodynamic benefits of the leader

We mainly focused on the hydrodynamic benefits of the follower in the present paper. Energy saving of the leader is also possible under specific spatiotemporal patterns (see Supplementary Fig. 7**c**). However, the rule behind this is very complicated. This may due to the complexity of turbulence in 3D and the high Reynolds number[16]. Considering the leader and follower as a unit system, energy saving also occurs (see Supplementary Fig. 7**d**). However the rule of energy saving for the follower contributes much more. This might indicates that a follower saves much more energy when compared to the leader (similar to the flying animal system[44,45]).

The frequency of occurrence of real fish adopting VPM to save energy

We tested the predictions of our analytical model in experiments with goldfish. While we found strong evidence that the follower exhibits vortex phase matching to save energy, this does not mean fish always, or only, follow this mechanism[35]. Similarly to previous observations, the occurrence of fish considering energy saving with VPM is moderate (see Supple-

mentary Fig. 23). This is likely due to the fact that, as discussed above, social information is also important for real fish[43,46]. Furthermore, fish may have evolved other strategies to save energy, such as swimming near the substratum[47], burst and gliding swimming patterns[48] and extracting energy by pectoral fin movements[49].

Responding to the local flow

When the lateral line is inhibited and we impair visual information by only applying infrared illumination[50], fish are still able to undulate their body following VPM. This indicates that other sensors, such as mechanosensor[51–53], tactile[54–56], and perhaps vestibular[57], may also contribute to sensing the local flow, and/or fish may respond passively to flow[13,20,34].

References

[1] Videler, J. & Hess, F. Fast continous swimming of two pelagic predators, saithe (*Pollachius virens*) and mackerel (*Scomber scombrus*) a kinematic analysis. *J. Exp. Biol.* **109**, 209–228 (1984).

[2] Ijspeert, A. J., Crespi, A., Ryczko, D. & Cabelguen, J. M. From swimming to walking with a salamander robot driven by a spinal cord model. *Science* **315**, 1416–1420 (2007).

[3] Li, L., Wang, C. & Xie, G. A general CPG network and its implementation on the microcontroller. *Neurocomputing* **167**, 299–305 (2015).

[4] Breder, C. M. Equations descriptive of fish schools and other animal aggregations. *Ecology* **35**, 361–370 (1954).

[5] Partridge, B. L. The structure and function of fish schools. *Sci. Am.* **246**, 114–122 (1982).

[6] Tian, R. *et al.* CFD based parameter tuning for motion control of robotic fish. *Bioinspir. Biomim.* **15**, 026008 (2020).

[7] Kulpa, M., Bak-Coleman, J. & Coombs, S. The lateral line is necessary for blind cavefish rheotaxis in non-uniform flow. *J. Exp. Biol.* **218**, 1603–1612 (2015).

[8] Bak-Coleman, J., Court, A., Paley, D. A. & Coombs, S. The spatiotemporal dynamics of rheotactic behavior depends on flow speed and available sensory information. *J. Exp. Biol.* **216**, 4011–4024 (2013).

[9] Berman, G. J., Choi, D. M., Bialek, W. & Shaevitz, J. W. Mapping the stereotyped behaviour of freely moving fruit flies. *J. Royal Soc. Interface* **11**, 20140672 (2014).

[10] Fisher, N. I. *Statistical analysis of circular data* (Cambridge University Press, 1995).

[11] Nadler, L. E., Killen, S. S., McClure, E. C., Munday, P. L. & McCormick, M. I. Shoaling reduces metabolic rate in a gregarious coral reef fish species. *J. Exp. Biol.* **219**, 2802–2805 (2016).

[12] Fish, F. E. & Lauder, G. V. Passive and active flow control by swimming fishes and mammals. *Annu. Rev. Fluid Mech.* **38**, 193–224 (2006).

[13] Zhu, X., He, G. & Zhang, X. Flow-mediated interactions between two self-propelled flapping filaments in tandem configuration. *Phys. Rev. Lett* **113**, 238105 (2014).

[14] Hemelrijk, C. K., Reid, D., Hildenbrandt, H. & Padding, J. T. The increased efficiency of fish swimming in a school. *Fish Fish.* **16**, 511–521 (2014).

[15] Peng, Z.-R., Huang, H. & Lu, X.-Y. Collective locomotion of two closely spaced self-propelled flapping plates. *J. Fluid Mech.* **849**, 1068–1095 (2018).

[16] Daghooghi, M. & Borazjani, I. The hydrodynamic advantages of synchronized swimming in a rectangular pattern. *Bioinspir. Biomim.* **10**, 056018 (2015).

[17] Schultz, W. W. & Webb, P. W. Power requirements of swimming: Do new methods resolve old questions? *Integr. Comp. Biol.* **42**, 1018–1025 (2002).

[18] Jia, L.-B. & Yin, X.-Z. Passive oscillations of two tandem flexible filaments in a flowing soap film. *Phys. Rev. Lett* **100**, 228104 (2008).

[19] Ristroph, L. & Zhang, J. Anomalous hydrodynamic drafting of interacting flapping flags. *Phys. Rev. Lett* **101**, 194502 (2008).

[20] Becker, A. D., Masoud, H., Newbolt, J. W., Shelley, M. & Ristroph, L. Hydrodynamic schooling of flapping swimmers. *Nat. Commun.* **6**, 97 (2015).

[21] Dewey, P. A., Quinn, D. B., Boschitsch, B. M. & Smits, A. J. Propulsive performance of unsteady tandem hydrofoils in a side-by-side configuration. *Phys. Fluids* **26**, 041903 (2014).

[22] Ramananarivo, S., Fang, F., Oza, A., Zhang, J. & Ristroph, L. Flow interactions lead to orderly formations of flapping wings in forward flight. *Phys. Rev. Fluids* **1**, 071201 (2016).

[23] Quinn, D. B., Lauder, G. V. & Smits, A. J. Scaling the propulsive performance of heaving flexible panels. *J. Fluid Mech.* **738**, 250–267 (2014).

[24] Boschitsch, B. M., Dewey, P. A. & Smits, A. J. Propulsive performance of unsteady tandem hydrofoils in an in-line configuration. *Phys. Fluids* **26**, 051901 (2014).

[25] Maertens, A. P., Triantafyllou, M. S. & Yue, D. K. P. Efficiency of fish propulsion. *Bioinspir. Biomim.* **10**, 046013 (2015).

[26] Bao, Y. & Tao, J. J. Dynamic reactions of a free-pitching foil to the reverse Kármán vortices. *Phys. Fluids* **26**, 031704 (2014).

[27] Liu, G. *et al.* Computational analysis of vortex dynamics and performance enhancement due to body–fin and fin–fin interactions in fish-like locomotion. *J. Fluid Mech.* **829**, 65–88 (2017).

[28] Weihs, D. Hydromechanics of fish schooling. *Nature* **241**, 290–291 (1973).

[29] Deng, J. & Shao, X.-M. Hydrodynamics in a diamond-shaped fish school. *J. Hydrodyn. Ser. B* **18**, 438–442 (2006).

[30] Park, S. G. & Sung, H. J. Hydrodynamics of flexible fins propelled in tandem, diagonal, triangular and diamond configurations. *J. Fluid Mech.* **840**, 154–189 (2018).

[31] Han, J., Zhang, Y. & Chen, G. Effects of individual horizontal distance on the three-dimensional bionic flapping multi-wings in different schooling configurations. *Phys. Fluids* **31**, 041903 (2019).

[32] Oza, A. U., Ristroph, L. & Shelley, M. J. Lattices of Hydrodynamically Interacting Flapping Swimmers. *Phys. Rev. X* **9**, 041024 (2019).

[33] Maertens, A. P., Gao, A. & Triantafyllou, M. S. Optimal undulatory swimming for a single fish-like body and for a pair of interacting swimmers. *J. Fluid Mech.* **813**, 301–345 (2017).

[34] Newbolt, J. W., Zhang, J. & Ristroph, L. Flow interactions between uncoordinated flapping swimmers give rise to group cohesion. *Proc. Natl Acad. Sci. USA* **46**, 2419–2424 (2019).

[35] Partridge, B. L. & Pitcher, T. J. Evidence against a hydrodynamic function for fish schools. *Nature* **279**, 418–419 (1979).

[36] Katz, Y., Tunstrøm, K., Ioannou, C. C., Huepe, C. & Couzin, I. D. Inferring the structure and dynamics of interactions in schooling fish. *Proc. Natl Acad. Sci. USA* **108**, 18720–18725 (2011).

[37] Marras, S. *et al.* Fish swimming in schools save energy regardless of their spatial position. *Behav. Ecol. Sociobiol.* **69**, 219–226 (2014).

[38] Dong, G.-J. & Lu, X.-Y. Characteristics of flow over traveling wavy foils in a side-by-side arrangement. *Phys. Fluids* **19**, 057107 (2007).

[39] Verma, S., Novati, G. & Koumoutsakos, P. Efficient collective swimming by harnessing vortices through deep reinforcement learning. *Proc. Natl Acad. Sci. USA* **115**, 5849–5854 (2018).

[40] Ashraf, I. *et al.* Simple phalanx pattern leads to energy saving in cohesive fish schooling. *Proc. Natl Acad. Sci. USA* **114**, 9599–9604 (2017).

[41] Delcourt, J., Denoël, M., Ylieff, M. & Poncin, P. Video multitracking of fish behaviour: a synthesis and future perspectives. *Fish Fish.* **14**, 186–204 (2012).

[42] Gemmell, B. J., Colin, S. P., Costello, J. H. & Dabiri, J. O. Suction-based propulsion as a basis for efficient animal swimming. *Nat. Commun.* **6**, 159 (2015).

[43] Abrahams, M. V. & Colgan, P. W. Fish schools and their hydrodynamic function: a reanalysis. *Environ. Biol. Fishes* **20**, 79–80 (1987).

[44] Portugal, S. J. *et al.* Upwash exploitation and downwash avoidance by flap phasing in ibis formation flight. *Nature* **505**, 399–402 (2014).

[45] Voelkl, B. *et al.* Matching times of leading and following suggest cooperation through direct reciprocity during V-formation flight in ibis. *Proc. Natl Acad. Sci. USA* **112**, 2115–2120 (2015).

[46] Strandburg-Peshkin, A. *et al.* Visual sensory networks and effective information transfer in animal groups. *Curr. Biol.* **23**, R709–R711 (2013).

[47] Quinn, D. B., Lauder, G. V. & Smits, A. J. Flexible propulsors in ground effect. *Bioinspir. Biomim.* **9**, 036008 (2014).

[48] Fish, F. E., Fegely, J. F. & Xanthopoulos, C. J. Burst-and-coast swimming in schooling fish (*tNotemigonus crysoleucas*) with implications for energy economy. *Comp. Biochem. Physiol.* **100**, 633–637 (1991).

[49] Johansen, J. L., Vaknin, R., Steffensen, J. F. & Domenici, P. Kinematics and energetic benefits of schooling in the labriform fish, striped surfperch *Embiotoca lateralis*. *Mar. Ecol.-Prog. Ser.* **420**, 221–229 (2010).

[50] Palacios, A. G., Varela, F. J., Srivastava, R. & Goldsmith, T. H. Spectral sensitivity of cones in the goldfish, carassius auratus. *Vis. Res.* **38**, 2135–2146 (1998).

[51] Dickinson, M. H. How animals move: an integrative view. *Science* **288**, 100–106 (2000).

[52] Aiello, B. R., Westneat, M. W. & Hale, M. E. Mechanosensation is evolutionarily tuned to locomotor mechanics. *Proc. Natl Acad. Sci. USA* **114**, 4459–4464 (2017).

[53] Aiello, B. R., Hardy, A. R., Westneat, M. W. & Hale, M. E. Fins as mechanosensors for movement and touch-related behaviors. *Integr. Comp. Biol.* **84**, 2709 (2018).

[54] Lyon, E. On rheotropism in fishes. *Am. J. Physiol.* **12**, 149–161 (1904).

[55] Baker, C. F. & Montgomery, J. C. Lateral line mediated rheotaxis in the antarctic fish pagothenia borchgrevinki. *Polar Biol.* **21**, 305–309 (1999).

[56] Baker, C. & Montgomery, J. The sensory basis of rheotaxis in the blind Mexican cave fish, *Astyanax fasciatus*. *J. Comp. Physiol. A* **184**, 519–527 (1999).

[57] Pavlov, D. & Tyuryukov, S. The role of lateral-line organs and equilibrium in the behavior and orientation of the dace, *Leuciscus leuciscus*, in a turbulent flow. *J. Ichthyol* **33**, 45–45 (1993).
