# Supplementary material for: Vortex phase matching as a strategy for schooling in robots and in fish
Source: Nat Commun. 2020 Oct 26;11:5408. doi: 10.1038/s41467-020-19086-0 (PMC7588453; doi:10.1038/s41467-020-19086-0)
Supplement: Supplementary file 3 — Reporting Summary [file 41467_2020_19086_MOESM3_ESM.pdf]

nature research

Corresponding author(s): Mate Nagy, Guangming Xie, Iain Couzin

Last updated by author(s): Jun 5, 2020

Reporting Summary

Nature Research wishes to improve the reproducibility of the work that we publish. This form provides structure for consistency and transparency in reporting. For further information on Nature Research policies, see Authors & Referees and the Editorial Policy Checklist .

Statistics

For all statistical analyses, confirm that the following items are present in the figure legend, table legend, main text, or Methods section.

n/a	Confirmed	
☐	☒	The exact sample size (n) for each experimental group/condition, given as a discrete number and unit of measurement
☐	☒	A statement on whether measurements were taken from distinct samples or whether the same sample was measured repeatedly
☐	☒	The statistical test(s) used AND whether they are one- or two-sided<br>*Only common tests should be described solely by name; describe more complex techniques in the Methods section.*
☐	☒	A description of all covariates tested
☐	☒	A description of any assumptions or corrections, such as tests of normality and adjustment for multiple comparisons
☐	☒	A full description of the statistical parameters including central tendency (e.g. means) or other basic estimates (e.g. regression coefficient) AND variation (e.g. standard deviation) or associated estimates of uncertainty (e.g. confidence intervals)
☐	☒	For null hypothesis testing, the test statistic (e.g. F, t, r) with confidence intervals, effect sizes, degrees of freedom and P value noted<br>*Give P values as exact values whenever suitable.*
☒	☐	For Bayesian analysis, information on the choice of priors and Markov chain Monte Carlo settings
☒	☐	For hierarchical and complex designs, identification of the appropriate level for tests and full reporting of outcomes
☐	☒	Estimates of effect sizes (e.g. Cohen's d, Pearson's r), indicating how they were calculated

Our web collection on statistics for biologists contains articles on many of the points above.

Software and code

Policy information about availability of computer code

Data collection	Videos were collected using commercial code (Loopbio, Austria). Fish posture tracking is conducted by DeepPoseKit. Further steps of the data collection were performed using custom scripts written in Python (Python Software Foundation, 2018).
Data analysis	All the data analyses were performed using custom scripts written in MATLAB (MathWorks Inc., Natick, MA, USA) and Python (Python Software Foundation, 2018).

For manuscripts utilizing custom algorithms or software that are central to the research but not yet described in published literature, software must be made available to editors/reviewers. We strongly encourage code deposition in a community repository (e.g. GitHub). See the Nature Research guidelines for submitting code & software for further information.

Data

Policy information about availability of data

All manuscripts must include a data availability statement. This statement should provide the following information, where applicable:

- Accession codes, unique identifiers, or web links for publicly available datasets
- A list of figures that have associated raw data
- A description of any restrictions on data availability

All code and video data that support the findings of this study are available from the corresponding authors upon request.

Field-specific reporting

Please select the one below that is the best fit for your research. If you are not sure, read the appropriate sections before making your selection.

☐ Life sciences ☒ Behavioural & social sciences ☐ Ecological, evolutionary & environmental sciences

Behavioural & social sciences study design

All studies must disclose on these points even when the disclosure is negative.

Study description	quantitative experimental, comparative
Research sample	2 robotic fish and 32 random goldfish (Carassius auratus, around 20 cm) from a commercial supplier in Germany.
Sampling strategy	We determined sample size according to the three Rs principle (Replacement, Reduction and Refinement). A random test help us determine the effectiveness of sample size.
Data collection	Experiments on robotic fish were conducted in a low turbulence flow tank at the College of Engineering, Peking University. The experimental apparatus is around 2m-wide, 20m-long and 10m-high, and test area is around 0.4m-wide, 1m-long, 0.45m-deep.<br>Our robotic fish were placed in the flow tank and suspended from a six-axis control platform. The phase difference was controlled by initialising the movement of the follower robot after a specific time delay from the leader robot. The robot was powered at 6 Volts by a stabilised power supply (RIGOL DP832). We sampled the current required to power the fish at 5000 Hz using a current acquisition device from National Instruments (NI 9227). To minimise noise and error from initialisation effects, data were collected only after both 10 seconds had passed, and at least 5 full undulations had occurred, and we recorded the average the power cost (current x voltage) across 10 subsequent undulations. Each test was repeated 5 times and the efficiency coefficient was evaluated with average power cost of 5 repetitions.<br>During the robotic fish experiments, the flow speed of the flow tank was set to the swimming speed of the robot in static water (0.245 cm/s). In order to measure and remove baseline servo power costs, we conducted experiments both in air (suspended above the flow tank) and water. In total, we conducted 10,080 experiments with two robots swimming alone in air, alone in water and schooling in water with various phase differences ranging from 0 to 2pi with an interval of 0.2pi, front-back distance ranging from 0.22 to 1 body lengths (BL) with an interval of 0.022 BL and left-right distance ranging from 0.27 to 0.33 BL with an interval of 0.022 BL.<br><br>Experiments with goldfish were conducted in a flow tank (Loligo system, Tjele, Denmark). Before the experiments, we calibrated the flow speed with a vane wheel flow probe (Hontzsch, Germany). For each experiment, we randomly picked two fish, inhibited the lateral line if necessary and moved them to an acclimation container, which was slowly filled with water from the flow tank. After 30 minutes, and if the temperature difference between the flow tank and the acclimation container was less than 2 degrees, we transferred both fish to the flow tank. We left the fish inside the flow tank for another 30 minutes without flow. During this period, several snapshots were taken to measure body length of each fish. Flow speed was set according to the average body length of the fish and ranged from 1.2 BL/s (Body length/second) to 1.6 BL/s with an interval of 0.1 BL/s. White light was given for the intact (V+LL+) and lateral line impaired fish (V+LL-), and infra-red light (850nm) was given for the vision impaired intact (V-LL+) or both vision and lateral line impaired fish (V-LL-). We recorded the swimming of fish pairs in the flow tank from both bottom-view and side-view for 5 minutes with different flow speeds (in a randomised order) with 5 minute resting periods without flow in between.<br><br>No one was present during experiments, only the researchers were present for adding and moving animals from the experimental setup. The researchers were not blind to experimental condition and the study hypothesis during data collection.
Timing	All the robotic fish experiments were conducted during 20/11/2014~05/12/2014. All the real fish experiments were conducted during 10/12/2017 ~ 21/12/2017 and 23/07/2018~27/08/2018.
Data exclusions	Data were filtered before analysis according to the criteria described in the Supplementary methods-Data collection section. But no other data were excluded.
Non-participation	No participants dropped out.
Randomization	We randomly select two fish for each experiment.

Reporting for specific materials, systems and methods

We require information from authors about some types of materials, experimental systems and methods used in many studies. Here, indicate whether each material, system or method listed is relevant to your study. If you are not sure if a list item applies to your research, read the appropriate section before selecting a response.

Materials & experimental systems

n/a	Involved in the study	
☒	☐	Antibodies
☒	☐	Eukaryotic cell lines
☒	☐	Palaeontology
☐	☒	Animals and other organisms
☒	☐	Human research participants
☒	☐	Clinical data

Methods

n/a	Involved in the study	
☒	☐	ChIP-seq
☒	☐	Flow cytometry
☒	☐	MRI-based neuroimaging

Animals and other organisms

Policy information about studies involving animals; ARRIVE guidelines recommended for reporting animal research

Laboratory animals	goldfish (Carassius auratus, around 20 cm)
Wild animals	The study did not involve wild animals
Field-collected samples	The study did not involve samples collected from the field
Ethics oversight	All animal handling and experimental procedures were approved by Regierungsprasidium Freiburg, 35-9185.81/G-17/90.

Note that full information on the approval of the study protocol must also be provided in the manuscript.
