# Supplementary material for: Vortex phase matching as a strategy for schooling in robots and in fish
Source: Nat Commun. 2020 Oct 26;11:5408. doi: 10.1038/s41467-020-19086-0 (PMC7588453; doi:10.1038/s41467-020-19086-0)
Supplement: Supplementary file 4 — Description of Additional Supplementary Files [file 41467_2020_19086_MOESM4_ESM.pdf]

Descriptions of Additional Supplementary Files

Supplementary Movie 1

Description: Dye-visualisation of vortex shedding by free swimming robotic fish. Robotic fish produce clear reverse Kármán vortices, similar to those shed by real fish.

Supplementary Movie 2

Description: The six-axis test platform for robotic fish experiments. Six step motors control the 3-D position (xyz) of each robot allowing us to specify the left-right distance and front-back distance.

Supplementary Movie 3:

Description: Hydrogen bubble flow visualisation showing the vortex phase matching (VPM) that corresponds with maximal energy savings for the following robot. The follower moves its tail in the same direction as the induced flow of the vortices shed by the leader, which itself is determined by both the position and phase of the leader.

Supplementary Movie 4

Description: Deep-learning-based posture tracking of real fish. Phase, amplitude and frequency are extracted based on the tail tip movement (measured at the caudal peduncle) relative to head position.

Supplementary Movie 5

Description: Vortex phase matching in real fish. Theoretical predictions are based on our model by inputting amplitude, frequency, and front-back distance measured from the real fish positional and posture tracking.
